# Genome-wide analysis of miRNA and mRNA transcriptomes during amelogenesis

**DOI:** 10.1186/1471-2164-15-998

**Published:** 2014-11-19

**Authors:** Kaifeng Yin, Joseph G Hacia, Zhe Zhong, Michael L Paine

**Affiliations:** Center for Craniofacial Molecular Biology, Herman Ostrow School of Dentistry, University of Southern California, 2250 Alcazar Street, CSA103, Los Angeles, CA 90033 USA; Department of Biochemistry and Molecular Biology, Keck School of Medicine, University of Southern California, 2250 Alcazar Street, CSA140, Los Angeles, CA 90033 USA

**Keywords:** miRNA, Amelogenesis, Enamel maturation, Matrix mineralization, Bioinformatics

## Abstract

**Background:**

In the rodent incisor during amelogenesis, as ameloblast cells transition from secretory stage to maturation stage, their morphology and transcriptome profiles change dramatically. Prior whole genome transcriptome analysis has given a broad picture of the molecular activities dominating both stages of amelogenesis, but this type of analysis has not included miRNA transcript profiling. In this study, we set out to document which miRNAs and corresponding target genes change significantly as ameloblasts transition from secretory- to maturation-stage amelogenesis.

**Results:**

Total RNA samples from both secretory- and maturation-stage rat enamel organs were subjected to genome-wide miRNA and mRNA transcript profiling. We identified 59 miRNAs that were differentially expressed at the maturation stage relative to the secretory stage of enamel development (False Discovery Rate (FDR) < 0.05, fold change (FC) ≥ 1.8). In parallel, transcriptome profiling experiments identified 1,729 mRNA transcripts that were differentially expressed in the maturation stage compared to the secretory stage (FDR < 0.05, FC ≥1.8). Based on bioinformatics analyses, 5.8% (629 total) of these differentially expressed genes (DEGS) were highlighted as being the potential targets of 59 miRNAs that were differentially expressed in the opposite direction, in the same tissue samples. Although the number of predicted target DEGs was not higher than baseline expectations generated by examination of stably expressed miRNAs, Gene Ontology (GO) analysis showed that these 629 DEGS were enriched for ion transport, pH regulation, calcium handling, endocytotic, and apoptotic activities. Seven differentially expressed miRNAs (miR-21, miR-31, miR-488, miR-153, miR-135b, miR-135a and miR298) in secretory- and/or maturation-stage enamel organs were confirmed by *in situ* hybridization. Further, we used luciferase reporter assays to provide evidence that two of these differentially expressed miRNAs, miR-153 and miR-31, are potential regulators for their predicated target mRNAs, *Lamp1* (miR-153) and *Tfrc* (miR-31).

**Conclusions:**

In conclusion, these data indicate that miRNAs exhibit a dynamic expression pattern during the transition from secretory-stage to maturation-stage tooth enamel formation. Although they represent only one of numerous mechanisms influencing gene activities, miRNAs specific to the maturation stage could be involved in regulating several key processes of enamel maturation by influencing mRNA stability and translation.

**Electronic supplementary material:**

The online version of this article (doi:10.1186/1471-2164-15-998) contains supplementary material, which is available to authorized users.

## Background

Amelogenesis is the developmental process of dental enamel formation. Amelogenesis involves two major functional stages, secretory and maturation, and these stages are clearly demarcated by a transition zone in the continuously growing rodent incisor teeth [1]. The transition of ameloblasts from secretory to maturation stage, characterized by both morphological and functional changes, results in the formation of mature enamel with ordered crystallite structures. Gene dysregulation at any stage of amelogenesis can result in a group of hereditary conditions called *Amelogenesis Imperfecta* (AI) that adversely affect the structure and appearance of enamel [2–6]. Although researchers today have a very clear idea of the molecular activities that define secretory-stage amelogenesis [1], the molecular events that define enamel maturation remain understudied.

MicroRNAs (miRNA) are a class of small non-coding RNAs that regulate the expression of target genes by directly binding to their target mRNAs. To date, there are two functional studies that used the deletion of Dicer-1 to analyze miRNA function during tooth development [7, 8]. The epithelial deletion of Dicer-1, using the keratin 14 gene promoter-Cre recombinase combination (K14-Cre), does not induce embryonic tooth defects [8], whereas the earlier epithelial deletion of Dicer-1, triggered by Pitx2-Cre, or mesenchymal deletion under the control of Wnt1-Cre, led to a severe dental phenotype [7]. *In vitro* studies showed that miR-34a regulates human dental papilla cell differentiation by targeting NOTCH and TGF-beta signaling [9]. MiR-143 and miR-145 control odontoblast differentiation and dentin formation through KLF4 and OSX transcriptional factor signaling pathways [10].

Dynamic changes in miRNA levels have been observed during tooth development. Based on microarray profiling studies, 8 miRNAs have been identified to be both stage- and tissue-specific in murine tooth formation [8]. That is, miR-140, miR-31, miR-875-5p and miR-141 were expressed mainly during tooth morphogenesis identified at embryonic day 16 (E16), whereas miR-689, miR-720, miR-711 and miR-455 were prevalent at the cytodifferentiation stage (E18) [8]. A more recent study that combined both deep sequencing and microarray approaches to elucidate the miRNA expression profiles in the bud, cap, early bell and late bell stages of developing lower deciduous molars of miniature pigs identified 166 miRNAs expressed differentially across the four stages [11]. A subsequent bioinformatic prediction suggested that 18 of these miRNAs play key roles during tooth development, including let-7f, miR-128, miR-200b and miR-200c [11]. Two epithelial stem cell niches, located in the labial and lingual cervical loop regions, have been identified and shown to have different miRNA expression profiles [12]. Together these observations indicate that miRNAs are dynamically involved in tooth development by fine-tuning tooth morphogenesis and patterning, as well as terminal cell differentiation and tissue homeostasis.

To investigate the potential role of miRNA regulation in maturation-stage tooth development, we conducted genome-wide miRNA and mRNA transcript expression profiling analyses of secretory-stage and maturation-stage enamel organs obtained from rat incisors. We identified a group of stage-specific miRNAs and identified candidate gene targets based on bioinformatic prediction. Two maturation-stage-related genes, *Lamp1* and *Tfrc*, were verified by luciferase reporter assay to be the target genes of miRNA regulators. The results indicated a dynamic expression pattern of miRNAs during the transition from secretory-stage to maturation-stage enamel mineralization, and suggest that miRNAs can influence key processes of enamel maturation.

## Methods

### Animal dissection and total RNA isolation

All vertebrate animal studies complied with Institutional and Federal guidelines (Institutional Animal Care and Use Committee (IACUC) protocol number 11736). We used rat incisors as the source of total RNA, because the reference line that separates the secretory- and maturation-stage enamel organs along the enamel surface has been well established in rats [13, 14]. Four male Wistar Hannover rats, 4-week-old, weighing 100-110 g, were sacrificed for their mandibles. After being frozen and kept in liquid nitrogen overnight, the mandibles were subsequently lyophilized over the following 24 h. The bone encasing the enamel surface of incisors was then carefully removed and the exposed multicellular layer, which contains mostly the secretory- and maturation-stage enamel organs, was collected into separate RNase-free Eppendorf tubes. Dissection procedures followed previously described protocols [13, 15]. The total RNA including miRNA was extracted from secretory- and maturation-stage enamel organs using miRNeasy Mini Kit (Qiagen, Valencia, CA, USA). The enamel organs isolated from the four rats were fully processed and analyzed separately (RNA extraction, miRNA qPCR, whole genome array analysis and bioinformatics).

### Sample quality control by quantitative real-time PCR analysis

Using previously described methods, the expression of two stage-specific genes, *Odam* (most highly expressed during maturation stage) and *Enam* (most highly expressed during secretory stage), were checked by real-time PCR to ensure the accuracy of dissections and quality of total RNA collected from each individual sample [13, 16]. cDNA used for real-time PCR analysis of *Odam* and *Enam* was synthesized using miScript II RT Kit with miScript HiFlex Buffer (Qiagen). Real-time PCR reactions were performed with iQ SYBR® Green supermix (Bio-rad Life Sciences, Hercules, CA) and rat-specific primers (*Odam*-Forward: 5′-ATCAATTTGGATTTGTACCACA-3′, *Odam*-Reverse: 5′-CGTCGGGTTTATTTCAGAAGTGA-3′, *Enam*-Forward: 5′-TGCAGAAATACAGCTTCTCCT-3′, *Enam*-Reverse: 5′-CATTGGCATTGGCATGGCA-3′, *Actb*-Forward: 5′-AGTGTGACGTTGACATCCGTA-3′, *Actb*-Reverse: 5′-GCCAGGGCAGTAATCTCCTTCT-3′). For all RNA sample pairs (four rats each with secretory-stage and maturation-stage enamel organs) quantitative real-time PCR for *Odam* showed an increase in expression in maturation-stage by >130 fold, while *Enam* expression was clearly evident in the secretory stage, but negligible in the maturation stage. These results indicated that the samples were suitable to use for additional genome profiling experiments.

### Genome-wide miRNA and mRNA profiling analysis

Genome-wide miRNA profiling analysis was conducted based on the Rat miRNome miScript miRNA PCR Array (V16.0, 384-well; Qiagen), which profiles the expression of the 653 most abundantly expressed and best characterized miRNA sequences in the rat miRNA genome as annotated by the miRBase Release 16. cDNA was prepared using miScript II RT Kit with miScript HiSpec Buffer (Qiagen). MiScript SYBR Green PCR Kit (Qiagen) was used for the real-time PCR reactions on the miScript miRNA PCR Array and the real-time instrument was a LightCycler 480 (Roche Applied Science, IN, USA).

On the exact same RNA samples used for miRNA expression profiling analysis, we conducted genome-wide mRNA transcriptome analysis using RatRef-12-v1 Expression BeadChips (Illumina, Inc., San Diego, CA). This platform interrogates approximately 22,000 transcripts selected primarily from the National Center for Biotechnology Information (NCBI) RefSeq database (Release 16). All sample preparation, hybridization, BeadChips processing, and data acquisition were performed at the Southern California Genotyping Consortium (SCGC), according to the manufacturers’ recommended protocols.

MiRNA and mRNA expression profiling analyses were conducted on four animals, with each animal providing both secretory- and maturation-stage RNA samples. Each individual RNA sample was analyzed both for miRNA expression (triplicate technical replicates to ensure quality) and for mRNA expression. In total, 24 miRNA (8 individual samples in triplicate) and 8 mRNA global expression data sets were generated in this study. Original gene expression data files are available for download from the Gene Expression Omnibus (http://www.ncbi.nlm.nih.gov/geo/) under GEO accession record GSE59401.

### MiRNA and mRNA gene expression data processing

Raw miRNA expression data generated on miRNome miScript miRNA PCR Arrays were processed using SAS 9.2 statistical software. All raw data were acquired in the form of Ct values. The raw Ct values were normalized to the average Ct values of the six internal controls located in the last row of each 384-well plate. For the purpose of downstream analyses, we assigned a single expression score to each miRNA based on the average of data obtained from three replicates. All processed data from all 24-miRNA expression profiling experiments are provided in Additional file 1.

**Figure 1:**
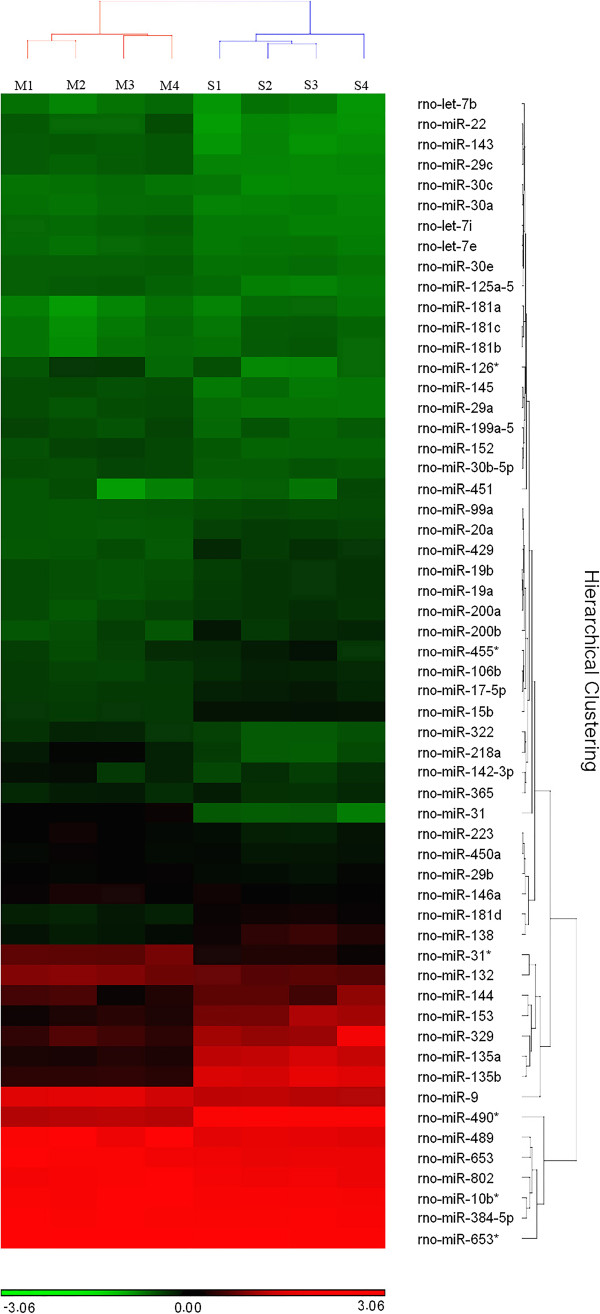
**Hierarchical clustering analysis of the expression levels of miRNAs with highest variability (Coefficient of Variance (CV) >0.15) across all samples.** All four pairs of samples are labeled in acronyms (Maturation-stage RNA sample 1–4: M1-4, Secretory-stage RNA sample 1–4: S1-4). The color scale from red to green indicates relative abundance of miRNAs from higher to lower.

Raw mRNA gene expression data generated using RatRef-12-v1 Expression BeadChips were processed using the R statistical package to produce normalized logarithm-transformed gene expression scores [15]. Processed data from all 8 mRNA expression profiling experiments are provided in Additional file 2. Differences in the expression levels of each mRNA and miRNA between secretory- and maturation-stage tooth development were evaluated using two-tail Student t test, and the type I error was controlled using the Benjamini Hochberg (B-H) method. In Additional file 3, we provide a summary of the numbers of differentially expressed mRNAs and miRNAs based on a variety of commonly used FC and FDR criteria. Hierarchical clustering analyses of mRNA and miRNA data sets were based on Euclidean distance and average linkage metrics and conducted using Partek® Genomics Suite version 6.5 (PGS) (Figure 1).

### Pathway analysis

**Figure 2:**
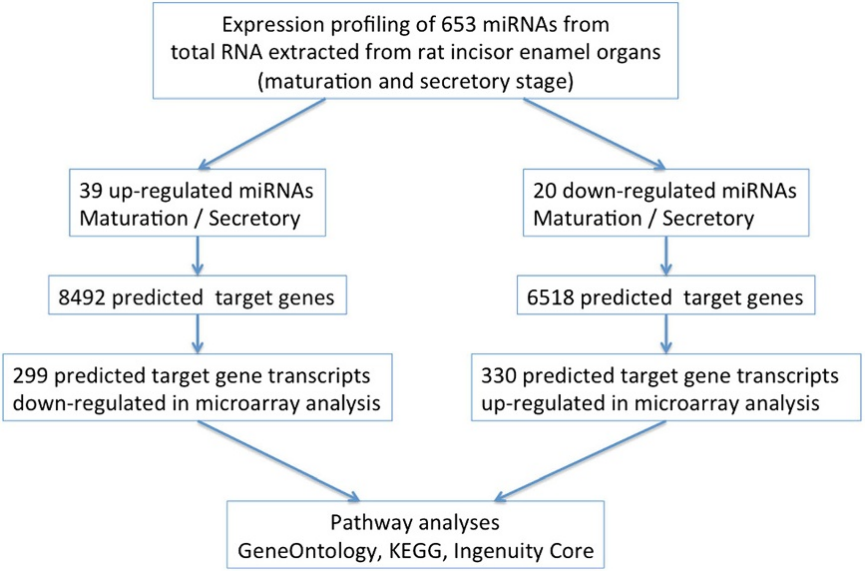
**Flow chart depicting the strategy used to select miRNA target genes for pathway analyses.**

We used Ingenuity Pathway Analysis (IPA) (Ingenuity Systems, Redwood City, CA) to predict gene targets for differentially expressed miRNAs identified by qPCR array analysis. In order to refine further the number of predicted targets for differentially expressed miRNAs, we used IPA to compare the list of predicted gene targets and the list of maturation stage-specific genes identified by mRNA transcriptome profiling on BeadChips. Genes in common between these two lists were uploaded to WebGestalt for Gene Ontology (GO) and KEGG (Kyoto Encyclopedia of Genes and Genomes) pathway analysis [17, 18] (Figure 2). The selection criteria for enriched GO categories were B-H adjusted *P* < 0.05 for Fisher’s exact test and minimum 5 genes in enriched categories. IPA also was used to analyze the functional relationships between differentially expressed miRNAs and these overlapping genes (Figure 2).

To begin to estimate baseline expectations of how miRNA level could influence mRNA levels in our system, we generated a list of stably expressed miRNAs present in both the secretory and maturation stages (herein defined as having raw Ct values ≤38 stages and whose relative expression levels showed no statistical differences between the two stages (FDR ≥ 0.05)). As above, we used IPA to predict mRNA targets for these stably expressed miRNAs and compared this list of predicted targets with the differentially expressed mRNAs identified through mRNA transcriptome profiling on BeadChips.

### In situ hybridization analysis of selected miRNAs expression

The mandibles were dissected out from euthanized Wistar Hannover rats (~100 g body weight,4 weeks-old), with the surrounding soft tissues removed. The hemimandibles were then fixed in 4% paraformaldehyde (PFA) at 4°C overnight, decalcified in 10% EDTA (pH 7.4) at 4°C for 10 weeks, and embedded in paraffin for sectioning. Sagittal sections of 7 μm were prepared and LNA-DIG miRNA detection probes, including U6 probes (positive control) and scrambled probes (Exiqon, Inc., MA, USA), were utilized for miRNA *in situ* hybridization. All the procedures for the *in situ* hybridization analyses were performed following the one-day protocol recommended by the manufacturers, with the probe concentration and substrate incubation time adjusted for each of tested probes individually. Information regarding probe sequences and the complete *in situ* hybridization protocol can be found on the website of Exiqon (http://www.exiqon.com).

### Cell culture and luciferase reporter assay

Mouse ameloblast-like LS8 cells [19, 20] were used as the host cells for exogenous miRNA mimics, miRNA inhibitors and luciferase reporter vectors in the luciferase reporter assay. LS8 cells were cultured in low-glucose DMEM medium (Gibco® Life Technologies, Grand Island, NY, USA) supplemented with 10% Fetal Bovine Serum (FBS) at 37°C in a 5% CO_2_ atmosphere. LS8 cells were seeded in 12-well cell culture plates to achieve a confluence of approximately 30% the day before transfection. Lipofectamine® LTX with Plus™ Reagent (Life Technologies) was diluted in FBS-free low-glucose DMEM medium at a concentration recommended by the manufacturers.

Luciferase reporter vectors containing 3′-UTR of target genes and/or miRNA mimics/inhibitors were mixed into diluted transfection reagents to form a transfection complex. Immediately before transfection, the cell culture medium was changed to FBS-free low-glucose DMEM medium. FBS-free low-glucose DMEM medium was removed 3 h after transfection, and cells were then incubated in low-glucose DMEM medium with 10% FBS. 48 h after transfection, cells were lysed using the passive lysis buffer provided in the Dual-Luciferase® Reporter Assay System (Promega, San Luis Obispo, CA, USA). Luciferase Assay Reagent (LARII) and Stop & Go reagent were then added to the cell lysates sequentially. The luciferase reporter activities were detected using a Turner Biosystems Luminometer TD-20/20 according to the manufacturers’ recommended protocol. Each luciferase reporter assay was conducted with triplicate technical replicates.

Two kinds of dual luciferase reporter vectors containing the 3′-UTR of different mouse-specific target genes (*Lamp1* and *Tfrc*) were purchased from GeneCopoeia (Catalog # MmiT029570-Lamp1, MmiT030401-Tfrc). MiRNA mimics and inhibitors for miR-153 and miR-31 were also mouse-specific and were obtained from Qiagen (Catalog # MSY0000163-miR-153 mimic, MIN0000163-miR-153 inhibitor, MSY0000538-miR-31 mimic, MIN0000538-miR-31 inhibitor). For verifying the relations between miR-153 (mature miRNA sequence: 5′-UUGCAUAGUCACAAAAGUGAUC-3′) and *Lamp1* expression, the setup of experimental groups involved LS8 cells: 1) transfected with luciferase reporter vector (3′-UTR of *Lamp1*); 2) co-transfected with miR-153 mimics and luciferase reporter vector (*Lamp1* 3′-UTR); 3) co-transfected with miR-153 inhibitors and luciferase reporter vector (*Lamp1* 3′-UTR). For miR-31 (mature miRNA sequence: 5′-AGGCAAGAUGCUGGCAUAGCUG-3′) and *Tfrc*, the experimental groups involved LS8 cells: 1) transfected with luciferase reporter vector (*Tfrc* 3′-UTR); 2) co-transfected by miR-31 mimics and luciferase reporter vector (*Tfrc* 3′-UTR); 3) co-transfected by miR-31 inhibitors and luciferase reporter vector (*Tfrc* 3′-UTR).

The amount of luciferase reporter vector was stabilized at 700 ng per transfection, to achieve optimal DNA transfection efficiency. The tested concentrations of miRNA mimics in final transfection complex (after being added into FBS-free cell culture medium in a 12-well plate) were 20pM, 60pM and 120pM, while the tested concentrations of miRNA inhibitors were 0.2nM, 0.6nM and 1.2nM (note that it is recommended by the manufacturers that the concentration of miRNA inhibitors should be ~10 times that of miRNA mimics).

Renilla luciferase activities were normalized to the firefly luciferase activities. For each verification experiment (miR-153 with *Lamp1* or miR-31 with *Tfrc*), two-tail Student t-test was used to detect the statistical differences in normalized luciferase activities between experimental groups 1 and 2, and between groups 1 and 3. In real-time PCR analyses of miRNA levels in LS8 cells following transfection, the raw Ct values were normalized to those of RNU6-2. The level of miRNA at each time point after transfection was calculated relative to the level of RNU6-2 using the ΔCt method. The significance level for all statistical analyses mentioned above was *P* < 0.05.

### Real-time PCR analysis of cellular miRNA levels

Prior to luciferase reporter assays, the levels of miR-153 and miR-31 in LS8 cells were detected using miRNA real-time PCR analysis at different time points: 0 h, 6 h, 24 h and 48 h following transfection by miRNA mimics or inhibitors. The levels of miR-153 and miR-31 were calculated relative to the level of RNA U6 Small Nuclear 2 (RNU6-2), as recommended by the manufacturers. The protocols for RNA extraction, cDNA synthesis and real-time PCR reactions were similar to the protocols of qPCR array analyses stated above. The mouse-specific primers of miR-153, miR-31 and RNU6-2 were purchased from Qiagen (Catalog # MS00011214-miR-153 primers, MS00001407-miR-31 primers, MS00033740-RNU6-2 primers), and the primer sequences were not disclosed by the manufacturers.

## Results

### miRNAs are differentially expressed in enamel organs as they transition from secretory stage to maturation stage in tooth development

The miRNA expression profiles of maturation-stage enamel development were compared to those of secretory-stage enamel development using total RNA samples obtained from the enamel organs of rat incisors. Although we considered multiple statistical cut-offs (Additional files 3 and 4), herein we assigned differential expression based on ≥1.8-FC and <5% FDR between the two developmental stages. This provided a robust set of differentially expressed miRNAs for follow-up analysis.

**Figure 3:**
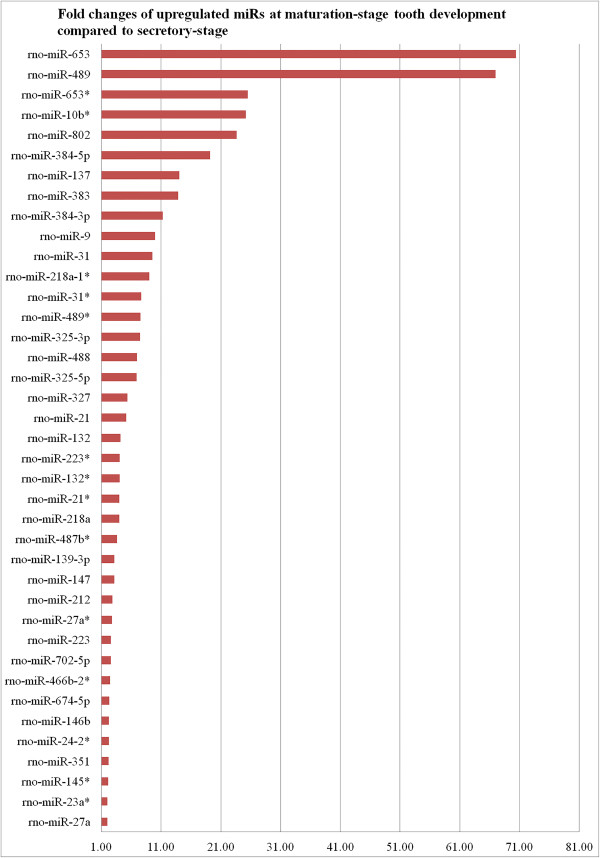
**Fold-changes of 39 up-regulated miRNAs (FDR < 0.05) at maturation stage relative to secretory stage based on miRNA qPCR array analysis.**

**Figure 4:**
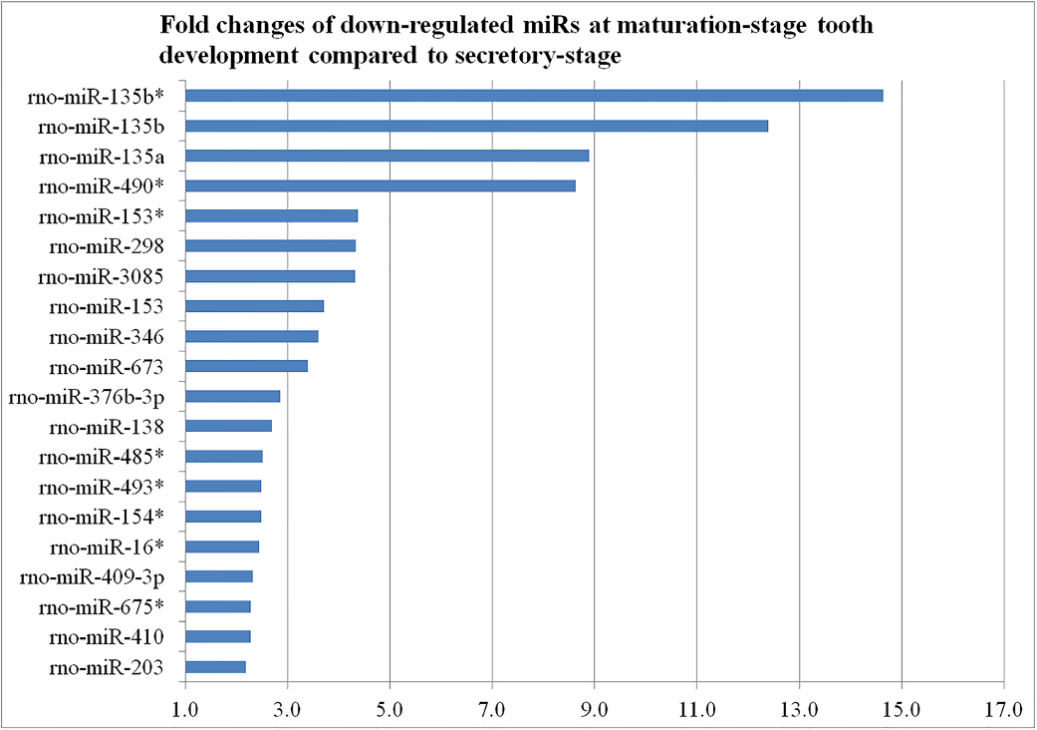
**Fold-changes of 20 down-regulated miRNAs (FDR < 0.05) at maturation stage relative to secretory stage based on miRNA qPCR array analysis.**

Hierarchical clustering analysis of the most variably expressed miRNAs across all samples showed that the maturation and secretory stages of enamel development have distinct miRNA expression profiles (Figure 1). All the maturation-stage samples and all the secretory-stage samples were placed into non-overlapping groups. A total of 59 out of 653 miRNAs were identified as being differentially expressed within maturation-stage enamel organs when compared to secretory-stage enamel organs. Among these 59 stage-specific miRNAs, 39 were up-regulated during maturation stage (relative to secretory stage) (Figure 3, Additional file 1) while 20 were down-regulated (Figure 4, Additional file 1). All raw Ct values and relevant statistics can be found in the supplemental information (Additional file 1).

### Differentially expressed genes are identified at mRNA level

The eight total RNA samples used for parallel genome-wide transcript profiling (i.e., four secretory-stage samples and four maturation-stage samples) were from the same samples as those used for miRNA expression profiling such that the two data sets (genome-wide transcript and miRNA expression profiling) could be matched with each other. To be consistent with our miRNA data analysis and also identify robust signals, we used the same criteria for differential gene expression as above (≥1.8-FC, <5% FDR) between the two developmental stages. A total of 1,729 genes were differentially expressed (701 down- and 1,028 up-regulated) in the maturation-stage enamel organs compared to the secretory-stage enamel organs (Additional file 2).

### Identifying differentially expressed genes that are predicted targets of the differentially expressed miRNAs

The strategy we used to identify targets of differentially expressed miRNAs is provided (Figure 2). The list of differentially expressed miRNAs was uploaded to IPA to generate predicted gene targets using integrated prediction algorithms (TargetScan, TarBase, miRecords and Ingenuity® Knowledge Base). A total of 8,492 unique candidate target genes were predicted to be regulated by the 39 up-regulated (maturation- relative to secretory-stage) miRNAs. Likewise, 6,518 unique candidate target genes were predicted to be regulated by the 20 down-regulated (maturation- relative to secretory-stage) miRNAs. In parallel, we used the same computational methods to predict 16492 unique gene targets for the 516 stably expressed miRNAs in these maturation and secretory stage samples (see Methods).

Next, we sought to refine the candidate relationships between predicted gene targets of differentially expressed miRNAs with our microarray-based measurements of mRNA levels. Approximately 5.8% (629/10,786) of the candidate target genes were differentially expressed in the expected direction in our analysis (i.e. over-expressed miRNA coincides with reduced expression of candidate target mRNA and vice versa). Next, we compared the list of predicted gene targets of up-regulated miRNAs with the observed list of down-regulated mRNAs during enamel maturation (Figure 2). A total of 299 observed down-regulated genes were identified as the potential targets for the 39 up-regulated miRNAs (Additional file 5). A total of 141 out of these 299 genes were predicted to be regulated by multiple miRNAs (Additional file 5; highlighted in bold). Conversely, we compared the predicted gene targets of down-regulated miRNAs with the observed list of up-regulated mRNAs during amelogenesis. A total of 330 up-regulated genes were identified as potential targets for the 20 down-regulated miRNAs (Additional file 6). Among these 330 genes, 105 were predicted to have multiple miRNA regulators (Additional file 6; highlighted in bold).

To place our observations in perspective, we compared the list of predicted gene targets of stably expressed miRNAs with the list of differentially expressed mRNAs (maturation- relative to secretory-stage). Approximately 8.1% of the candidate target mRNAs (1341 total: 828 up-regulated and 513 down-regulated) were identified to be the potential targets for the stably expressed miRNAs (Additional files 7, 8 and 9). Thus, it is not remarkable that 5.8% of the candidate target genes of differentially expressed miRNAs are differentially expressed in the expected direction. There are numerous potential reasons for this including (i) transcription factors will play a dominant role in influencing mRNA levels in accordance with our prior studies [13, 15] and (ii) the magnitude of the miRNA expression changes observed in our study where at least half of the protein-coding genes in the rodent genome were implicated as potential targets of differentially expressed miRNAs.

Thus, outside of translational regulation outside the scope of the current study, we propose that miRNAs more likely serves to fine-tune the levels of transcripts that are rapidly up-regulated or down-regulated during amelogenesis. For example, if the immediate up-regulation of a gene transcription is needed, then excessive mRNA may result, requiring immediate miRNA-targeting to reach optimal levels. In such cases, increased levels of specific targeting miRNA may be required to achieve this fine-tuning of highly up-regulated transcripts. To begin to address this issue, we identified 41 highly up-regulated mRNAs (≥5 fold, <5% FDR) during amelogenesis that could be paired with predicted targeting miRNAs that were also differentially expressed (≥1.8-fold, <5%FDR) during amelogenesis (Additional file 10). A subset of these transcripts was subject to follow-up experiments described below.

### Candidate miRNA regulated genes are enriched in key processes involved in enamel maturation

Next, we used various pathway analyses to explore relationships among the differentially expressed mRNAs that were candidate targets of differentially expressed miRNAs. GO analysis of the 330 up-regulated genes identified as potential targets for the 20 down-regulated miRNAs highlighted 120 enriched categories (Additional file 11). The categories most highly relevant to maturation-stage tooth development included: carboxylic acid transmembrane transporter activity (11 genes) (Table 1); ATPase activity, coupled to transmembrane movement of ions, phosphorylative mechanism (5 genes) (Table 2); endosome membrane (18 genes) (Table 3); lysosome (14 genes) (Table 4); calcium ion binding (27 genes) (Table 5); and cytokine activity (11 genes) (Table 6). GO analysis of the 299 down-regulated genes identified as potential targets for the 39 up-regulated miRNAs highlighted 82 enriched categories (Additional file 12). The categories that are seemingly most relevant to maturation-stage tooth development included: calcium ion transmembrane transporter activity (11 genes) (Table 7); and extracellular matrix part (8 genes), cation channel complex (7 genes), Golgi membrane (20 genes), GTPase activity (11 genes), regulation of cell-cell adhesion (7 genes) and cell junction (23 genes) (Additional file 12).

**Table 1:** **Genes enriched in the GO category “carboxylic acid transmembrane transporter activity” and their predicted miRNA regulators**

Human Gene symbol	mRNA Fold change	Predicted miRNA regulator(s)	miRNA Fold change
SLC1A1	46.7	rno-miR-298	-4.3
SLC6A8	5.6	rno-miR-135a	-8.9
SLC6A14	40.6	rno-miR-3085	-4.3
SLC16A6	3.1	rno-miR-135a/376b-3p	-8.9/-2.8
SLC22A5	1.8	rno-miR-138	-2.7
SLC23A2	4.3	rno-miR-138	-2.7
SLC25A15	2.6	rno-miR-138/3085	-2.7/-4.3
SLC26A1	39.8	rno-miR-138/298	-2.7/-4.3
SLC26A7	7.8	rno-miR-135a/153/298	-8.9/-3.7/-4.3
SLC36A1	1.9	rno-miR-298/3085/346	-4.3/-4.3/-3.6
SLC38A1	2.0	rno-miR-138/153	-2.7/-3.7

All fold changes represent the ratio of maturation to secretory phase expression values.

**Table 2:** **Genes enriched in the GO category “ATPase activity, coupled to transmembrane movement of ions, phosphorylative mechanism” and their predicted miRNA regulators**

Human Gene symbol	mRNA Fold change	Predicted miRNA regulator(s)	miRNA Fold change
ATP2A2	2.2	rno-miR-298	-4.3
ATP1B2	2.2	rno-miR-298/410	-4.3/-2.3
ATP1B3	2.4	rno-miR-153	-3.7
ATP6V1C1	2.5	rno-miR-135a/153	-8.9/-3.7
ATP6V1E1	3.0	rno-miR-135a	-8.9

All fold changes represent the ratio of maturation to secretory phase expression values.

**Table 3:** **Genes enriched in the GO category “endosome membrane” and their predicted miRNA regulators**

Human Gene symbol	mRNA Fold change	Predicted miRNA regulator(s)	miRNA Fold change
ATP6V0A1	2.2	rno-miR-138	-2.7
CD68	4.6	rno-miR-135a	-8.9
CFTR	4.9	rno-miR-298/3085/376b-3p	-4.3/-4.3/-2.8
ECE1	2.8	rno-miR-138	-2.7
EHD3	4.6	rno-miR-138/153	-2.7/-3.7
EHD4	3.3	rno-miR-376b-3p	-2.8
FCGR1A	1.8	rno-miR-3085	-2.7
LAMP1	3.2	rno-miR-153	-3.7
MYD88	2.4	rno-miR-138/298/3085/135a	-2.7/-4.3/-4.3/-8.9
PARM1	11.1	rno-miR-203	-2.2
PMEPA1	4.9	rno-miR-410	-2.3
RAB21	1.9	rno-miR-410	-2.3
SLC11A2	3.0	rno-miR-203	-2.2
SLC26A7	7.8	rno-miR-135a/153/298	-8.9/-3.7/-4.3
TFRC	28.6	rno-miR-490*	-8.6
TMBIM1	5.5	rno-miR-3085	-4.3
VPS37B	2.1	rno-miR-3085/376b-3p	-4.3/-2.8
ZNRF2	1.9	rno-miR-153	-3.7

All fold changes represent the ratio of maturation to secretory phase expression values.

**Table 4:** **Genes enriched in the GO category “lysosome” and their predicted miRNA regulators**

Human Gene symbol	mRNA Fold change	Predicted miRNA regulator(s)	miRNA Fold change
BGN	2.3	rno-miR-3085	-4.3
CD68	4.6	rno-miR-135a	-8.9
CTSS	2.0	rno-miR-203	-2.2
FMOD	3.5	rno-miR-203/138	-2.2/-2.7
LAMP1	3.2	rno-miR-153	-3.7
P2RY2	2.4	rno-miR-135a	-8.9
PDGFRB	2.8	rno-miR-3085	-4.3
PON2	1.9	rno-miR-376b-3p	-2.8
SDC3	1.8	rno-miR-138	-2.7
SLC11A2	3.0	rno-miR-203	-2.2
SLC36A1	1.9	rno-miR-298/3085/346	-4.3/-4.3/-3.6
STS	2.3	rno-miR-138	-2.7
TMBIM1	5.5	rno-miR-3085	-4.3
ZNRF2	1.9	rno-miR-153	-3.7

All fold changes represent the ratio of maturation to secretory phase expression values.

**Table 5:** **Genes enriched in the GO category “calcium ion binding” and their predicted miRNA regulators**

Human Gene symbol	mRNA Fold change	Predicted miRNA regulator(s)	miRNA Fold change
ANXA8L2	5.3	rno-miR-298/3085	-4.3/-4.3
ATP2A2	2.2	rno-miR-298	-4.3
BMP1	2.0	rno-miR-138	-2.7
CDH13	2.0	rno-miR-153	-3.7
CDH17	2.2	rno-miR-298	-4.3
CHP1	1.8	rno-miR-135a/298	-8.9/-4.3
CIB2	4.2	rno-miR-346/153	-3.6/-3.7
DSG2	2.0	rno-miR-153	-3.7
DUOX1	2.4	rno-miR-298	-4.3
EFHD2	2.8	rno-miR-138/153	-2.7/-3.7
EHD3	4.6	rno-miR-138/153	-2.7/-3.7
EHD4	3.3	rno-miR-376b-3p	-2.8
FAT3	2.9	rno-miR-153/203/3085	-3.7/-2.2/-4.3
FCN1	2.6	rno-miR-3085	-4.3
GCH1	5.0	rno-miR-490*	-8.6
LCP1	3.0	rno-miR-135a	-8.9
MAN1A1	4.5	rno-miR-135a/3085	-8.9/-4.3
MEGF6	2.8	rno-miR-135a/3085	-8.9/-4.3
MMP14	1.9	rno-miR-298/3085/410	-4.3/-4.3/-2.3
PLCB3	2.1	rno-miR-298	-4.3
RUNX1	2.2	rno-miR-298/3085/410/135a	-4.3/-4.3/-2.3/-8.9
RYR3	2.1	rno-miR-153	-3.7
SCUBE1	2.4	rno-miR-298	-4.3
SPARCL1	2.6	rno-miR-153	-3.7
STAT3	2.3	rno-miR-410	-2.3
STIM2	3.8	rno-miR-153/154*	-3.7/-2.5
SULF2	2.3	rno-miR-138	-2.7

All fold changes represent the ratio of maturation to secretory phase expression values.

**Table 6:** **Genes enriched in the GO category “cytokine activity” and their predicted miRNA regulators**

Human Gene symbol	mRNA Fold change	Predicted miRNA regulator(s)	miRNA Fold change
BMP1	2.0	rno-miR-138	-2.7
CCL19	24.7	rno-miR-298	-4.3
CSF1	2.1	rno-miR-3085	-4.3
CX3CL1	15.6	rno-miR-298	-4.3
GAB1	1.9	rno-miR-153/376b-3p/410	-3.7/-2.8/-2.3
IL1B	2.0	rno-miR-3085	-4.3
INHBA	4.8	rno-miR-135a	-8.9
TNFSF11	2.4	rno-miR-3085/410	-4.3/-2.3
TNFSF13	2.4	rno-miR-298	-4.3
WNT5A	3.0	rno-miR-410	-2.3
VEGFA	3.2	rno-miR-203/410	-2.2/-2.3

All fold changes represent the ratio of maturation to secretory phase expression values.

**Table 7:** **Genes enriched in the GO category “calcium ion transmembrane transporter activity” and their predicted miRNA regulators**

Human Gene symbol	mRNA Fold change	Predicted miRNA regulator(s)	miRNA Fold change
ATP2B1	-5.5	rno-miR-223/27a/384-5p/488/489	2.6/2.0/19.2/6.9/67.0
ATP2B3	-3.3	rno-miR-351	2.2
CACFD1	-1.8	rno-miR-137	14.1
CACNA1D	-2.0	rno-miR-137/384-5p/489	2.2/19.2/67.0
CACNA1G	-1.8	rno-miR-137/702-5p	14.1/2.6
CACNB2	-4.1	rno-miR-351/137/27a/384-5p/31/9	2.2/14.1/2.0/19.2/9.5/ 10.0
CACNB3	-1.9	rno-miR-351	2.2
CHRNA10	-5.5	rno-miR-351	2.2
NCS1	-2.7	rno-miR-223	2.6
SLC24A3	-2.4	rno-miR-137	14.1
SLC8A3	-3.8	rno-miR-21/489	5.2/67.0

All fold changes represent the ratio of maturation to secretory phase expression values.

**Figure 5:**
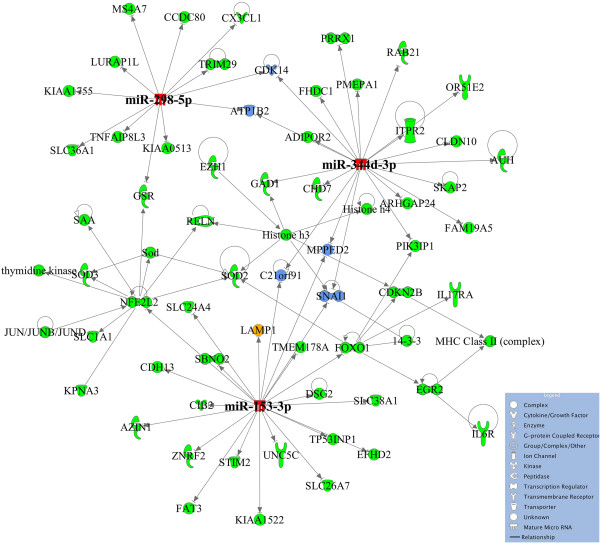
**Highest scoring miRNA-mRNA interaction network, “Cellular Movement, Neurological Diseases, Organismal Injury and Abnormalities.”** Figure was generated based on IPA software analysis of up-regulated miRNAs (maturation/secretory (M/S)) and their predicted gene target candidates that were down-regulated (M/S) with a top score of 110 and involvement of 62 molecules. The miRNA hubs, the genes that have multiple potential miRNA regulators, and the experimentally validated genes are colored red, blue and orange, respectively. The remaining genes in the network are colored green.

We also conducted KEGG analysis and IPA Core Analysis for each group of differentially expressed genes with and without involvement of miRNAs (Additional files 13, 14, 15, 16, 17, 18, 19, 20 and 21). In general, we did not find noteworthy categories relevant to maturation-stage tooth development. Nevertheless, we did find KEGG pathways, including calcium signaling, ECM-receptor interaction, lysosome and endocytosis, that could be relevant to enamel maturation (Additional files 13 and 14), and several IPA canonical pathways that are highly conserved during amelogenesis (Additional files 15, 16, 17 and 18). The IPA-derived interaction networks of differentially expressed miRNAs and their predicted gene targets showed clearly that miRNAs are regulatory hubs in these enriched functional pathways (Figure 5). In addition, several genes were targets of multiple miRNAs and served to connect these hubs. The details regarding the miRNA-mRNA interaction networks are provided in the (Additional files 15, 16, 17, 18, 19, 20 and 21).

### The expression of stage-specific miRNAs confirmed by in situ hybridization

**Figure 6:**
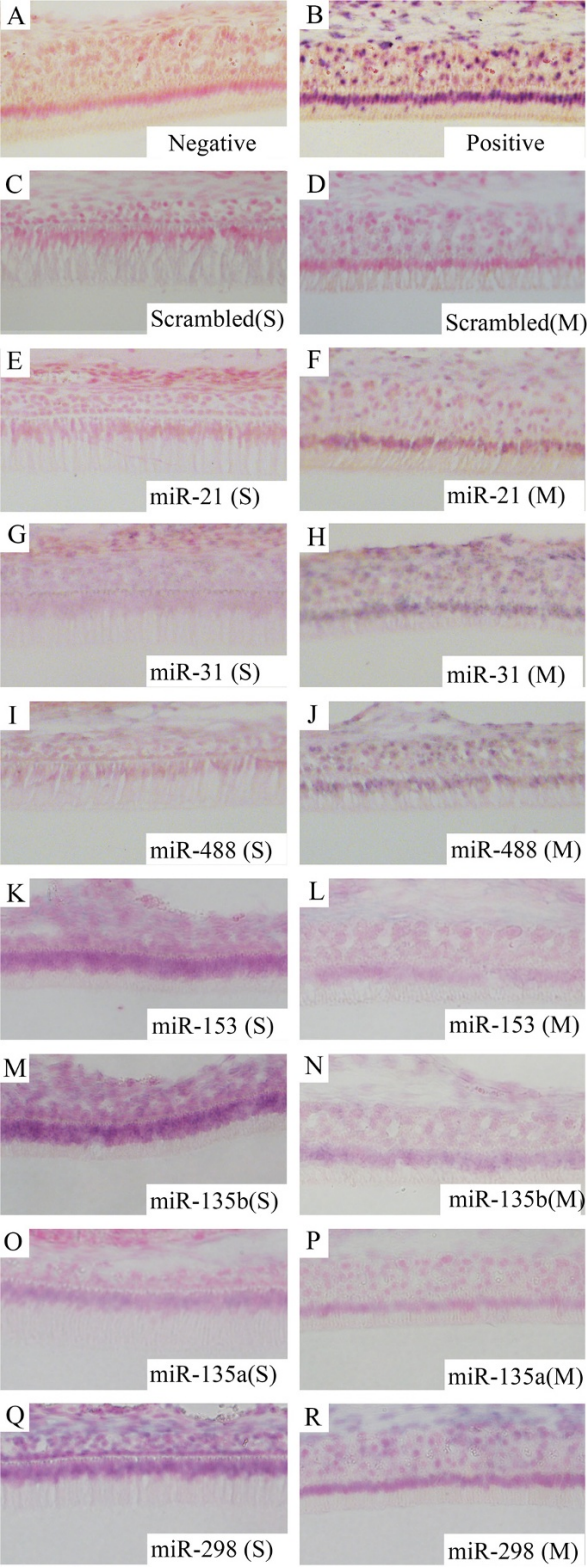
**Expression patterns of seven miRNAs in secretory- and maturation-stage enamel organs as shown by** ***in situ*** **hybridization. (A)** Negative control. Samples were incubated without any miRNA detection probes. **(B)** Positive control. U6 detection probes were used for incubation. **(C)** Scrambled control for secretory-stage enamel organ. **(D)** Scrambled control for maturation-stage enamel organ. **(E)**, **(G)**, **(I)**, **(K)**, **(M)**, **(O)** and **(Q)** The expression of miR-21, miR-31, miR-488, miR-153, miR-135b, miR-135a and miR-298 in secretory-stage enamel organ. MiR-21, miR-31 and miR-488 are down-regulated, while miR-153, miR-135b, miR-135a and miR-298 are up-regulated in secretory-stage enamel formation compared to maturation-stage. **(F)**, **(H)**, **(J)**, **(L)**, **(N)**, **(P)** and **(R)** The expression of miR-21, miR-31, miR-488, miR-153, miR-135b, miR-135a and miR-298 in maturation-stage enamel organ, showing altered maturation-stage expression patterns. Images shown at 20× magnification. S, secretory stage. M, maturation stage.

LNA-DIG miRNA probes were used to detect miRNA expression in enamel organs along the enamel surface of rat incisors (Figure 6). Mandible section slides stained with U6 LNA-DIG served as the positive controls (Figure 6B) and scrambled controls showed little background (Figure 6C and D). The expression patterns of seven selected miRNAs (miR-21, miR-31, miR-488, miR-153, miR-135b, miR-135a and miR-298) were examined (Figure 6E-R). *In situ* hybridization analyses of miR-21, miR-31 and miR-488 generated higher signal intensities in maturation-stage enamel organ cells than in secretory-stage enamel organ cells (Figures 6E and 4J), and this data correlates well (same directional change) with the miRNA qPCR array data/fold increase (i.e., miR-21, miR-31, miR-488 increased by 5.2, 9.5 and 6.9 fold, respectively) (Additional file 1). In contrast, as seen in the *in situ* slides, there was a decrease in the expression levels of miR-153, miR-135b, miR-135a and miR-298 from secretory-stage to maturation-stage tooth development (Figure 6K-R). This trend of expression correlates well with the miRNA qPCR array data (i.e., miR-153, miR-135b, miR-135a and miR-298 changed by -3.7, -12.4, -8.9 and -4.3 fold, respectively) (Additional file 1).

### Lamp1 and Tfrc are potential gene targets of miRNA regulators

According to previous bioinformatic studies, miR-153 is a predicted regulator of LAMP1, while miR-490* is a predicted regulator of TFRC (Table 3, Additional file 6). However, these target predictions for differentially expressed miRNAs were based mainly on human genome predictions; thus, we searched TargetScan (http://www.targetscan.org/) for the predicted miRNA regulators of these two genes within the mouse genome. We found that the prediction of miR-153 as the regulator of LAMP1/*Lamp1* was consistent between the human and mouse genomes. However, miR-490* was not listed as one of the predicted regulators for mouse-specific *Tfrc*. One of the miRNAs identified as being highly up-regulated in maturation-stage enamel organ cells is miR-31 (~10 fold increase when compared to secretory-stage), and bioinformatic prediction identifies miR-31 as a potential regulator of *Tfrc* in all vertebrate genomes. As a result, we decided to use miR-31 for subsequent *in vitro* verification that Tfrc could be subjected to miRNA regulation at some level in the mouse genome.

**Figure 7:**
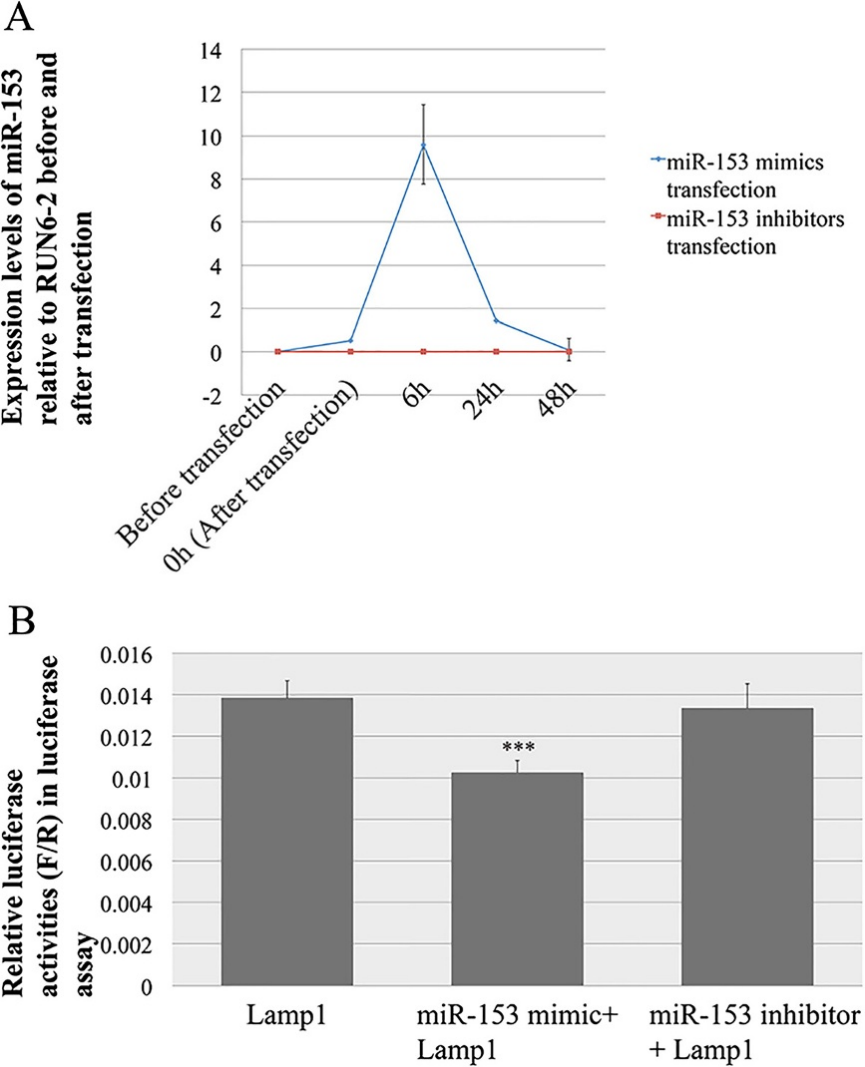
**Lamp1 is the potential target of miR-153. (A)** The expression levels of miR-153 in LS8 cells before and after transfection with miR-153 mimics (blue) and inhibitors (red). **(B)** Relative luciferase reporter activities in luciferase reporter assay validating the interaction of miR-153 with 3′-UTR of *Lamp1*. Luciferase activities are presented in ratios of Firefly (F) to Renilla (R) luciferase reporter activities. For the data presented, the amount of luciferase reporter vector used was 700 ng, and the concentrations of miR-153 mimics and inhibitors in final transfection complex were 20 pM and 0.2 nM, respectively.

**Figure 8:**
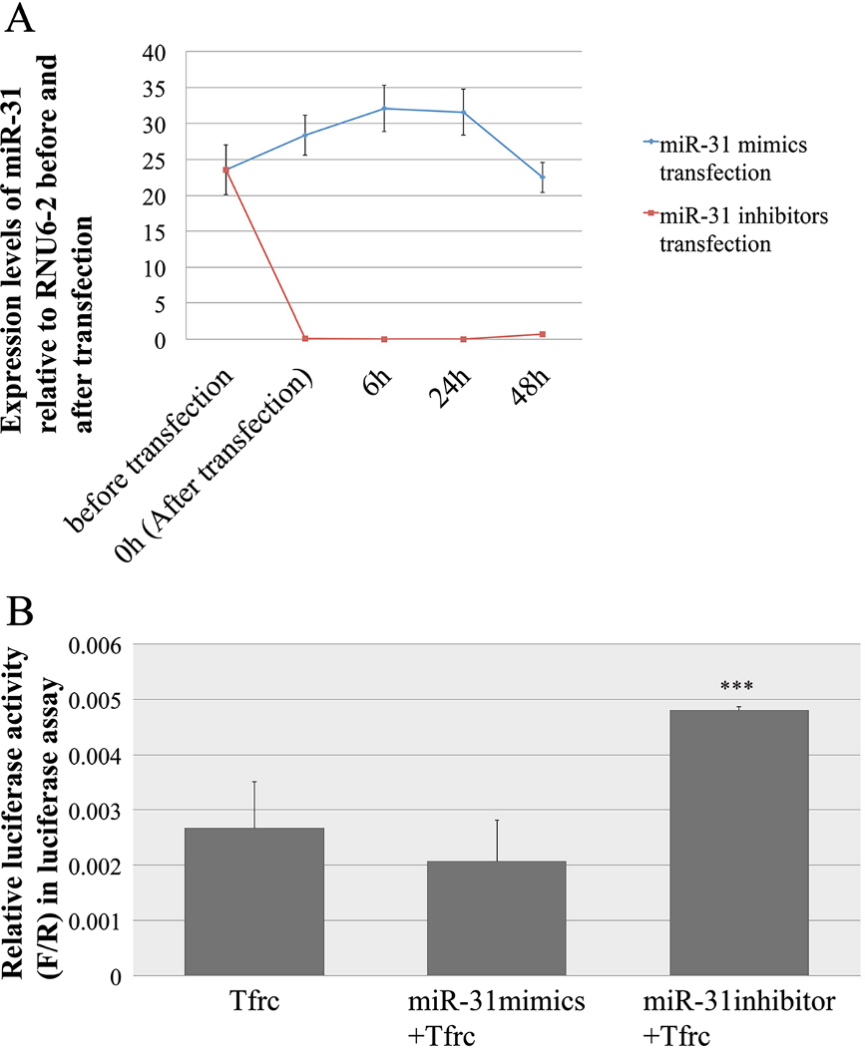
**Tfrc is the potential target of miR-31. (A)** The expression levels of miR-31 in LS8 cells before and after transfection with miR-31 mimics (blue) and inhibitors (red). **(B)** Relative Luciferase reporter activities in luciferase reporter assay validating the interaction of miR-31 with 3′-UTR of *Tfrc*. For the data presented, the amount of luciferase reporter vector used was fixed at 700 ng, and the concentrations of miR-31 mimics and inhibitors in final transfection complex were 20 pM and 0.2 nM respectively.

The expression levels of miR-153 and miR-31 in LS8 cells at different time points, following the transfection of corresponding miRNA mimics or inhibitors, were first checked separately using quantitative real-time PCR. Before LS8 cells were transfected by miR-153 mimics, the expression of miR-153 could not be detected (Figure 7A). During the first 6 h after transfection with miR-153 mimics, there was a sharp increase in the intracellular level of miR-153, which decreased continuously from 6 h through 48 h. At 48 h, the level of miR-153 returned to the original, non-detectable level. Because there was almost no endogenous miR-153 expression, miR-153 inhibitors did not change the intracellular level of miR-153, which remained at zero throughout the experiment (Figure 7A). By comparison, a higher level of intracellular miR-31 was detected in LS8 cells before transfection with miR-31 mimics, indicating that miR-31 was intrinsically expressed in LS8 cells (Figure 8A). The changes in the expression of intracellular miR-31 were relatively subtle following transfection with miR-31 mimics, while the introduction of miR-31 inhibitors repressed the intracellular miR-31 almost immediately (within 10 min) after transfection. The intracellular miR-31 level following transfection with the miR-31 inhibitors remained undetectable for the 48 h of observation.

In the luciferase reporter assay, exogenously introduced miR-153 mimics caused a decrease of 24.6% in the luciferase reporter activities (*P* = 0.007) (Figure 7B), indicating a direct interaction between the miR-153 mimics and the 3′-UTR of exogenously added *Lamp1* (contained in luciferase reporter vector). Introduction of miR-153 inhibitors into *Lamp1* 3′-UTR transfected LS8 cells failed to cause statistically significant changes in luciferase reporter activities (*P* = 0.776) (Figure 7B). This is consistent with the array analyses, in which miR-153 was down-regulated, and as a direct result *Lamp1* was up-regulated, during enamel maturation.

Exogenously added miR-31 mimics showed a small inhibitory effect, however it was not statistically significant (*P* = 0.284) (Figure 8B). This may suggest that the initial levels of endogenous miR-31 are so high that the targeting activity of exogenous miR-31 mimics on the 3′-UTR of *Tfrc* is minimal. When miR-31 inhibitors were added to *Tfrc* 3′-UTR LS8 cells there was a 77.8% increase in luciferase reporter activities (*P* = 0.008) (Figure 8B). Taken together these results would suggest that the *Tfrc* 3′-UTR luciferase vector is functional, and that the miR-31 mimics effectively target the *Tfrc* transcript. This data on miR-31 directed *Tfrc* transcript down-regulation is also consistent with the gene expression microarray analyses, in which both *Tfrc* and miR-31 were up-regulated during enamel maturation. That is, *Tfrc* is up-regulated by approximately 30 fold during maturation (Additional file 6), which is an extreme change in expression, and may require some “fine-tuning” and downward adjustments. The most immediate response from cells to fine-tune this *Tfrc* transcriptional up-regulation may be to activate processes that target *Tfrc* mRNAs for degradation, which may include gene up-regulation of miR-31 transcription.

Clearly, relating cell culture-based experiments to *in vivo*-derived data poses problems for interpretation, but these data sets for *Lamp1* and miR-153 interactions, as well as for *Tfrc* and miR-31 interactions, warrant further investigation.

## Discussion

The involvement of miRNAs in tooth development was first suggested in 2008 [21]. Since then, studies investigating miRNA expression profiles during amelogenesis have covered mainly the early developmental stages of odontogenesis prior to the secretory and maturation stages of amelogenesis [8, 11, 21]. The bioinformatics analyses from these earlier murine and porcine studies have suggested that key roles of these stage- and/or tooth-specific miRNAs relate primarily to cell differentiation, tooth morphogenesis and patterning [8, 11, 21]. In our study, we conducted genome-wide miRNA expression profiling analysis in secretory- and maturation-stage enamel organs obtained from rats, and identified 59 (out of 653) miRNAs that were differentially expressed between secretory- and maturation-stage tooth development. This is consistent with the dynamic expression pattern of miRNAs during tooth formation across species, as noted in prior studies [8, 11, 21], indicating possible regulatory roles of miRNAs in the later stages (enamel organ maturation) of enamel development.

Identifying target genes for miRNAs is essential to connect the stage-specific miRNA regulators to biological functions. Computational prediction provides a tool to generate list of candidate miRNA target genes [22–26]. However, the list of predicted genes for miRNAs can be intimidating (herein, over ten thousand), making it impractical for further investigation. To refine the number of predicted target genes for the 59 differentially expressed miRNAs, we conducted a parallel alignment analysis between the list of predicted genes and the list of stage-specific genes identified by genome-wide transcript expression profiling. The alignments generated three workable lists of predicted genes for the secretory- and maturation-stage-specific miRNAs (Additional files 5, 6 and 19). These were up-regulated miRNAs and their potential down-regulated mRNA targets (39 and 299 respectively; Additional file 5); down-regulated miRNAs and their potential up-regulated mRNA targets (20 and 330 respectively; Additional file 6); and up-regulated miRNAs and their most highly up-regulated potential mRNA targets (15 and 41 respectively; Additional file 19). Recent studies demonstrated that a majority (>80%) of mammalian miRNAs affect their gene targets at the mRNA level by decreasing the stability of target mRNAs [27] and the method of pairing inversed expression profiles of miRNAs and mRNAs has been successfully used in previous miRNA expression profiling studies [28–35]. Nevertheless, we were still at risk of including false positive candidates and/or excluding valid candidates from the final lists due to the intrinsic complexity of miRNA regulation [27, 36–39]. Another potential problem with miRNA target prediction is that, because the genetic information about miRNA target prediction is relatively sparse in rodents compared to humans, all procedures and software involved in target prediction are based on homologous human miRNAs, that may also increase the false discovery and false negative rates.

The functions of ameloblasts during maturation-stage tooth development include matrix turnover, calcium handling, pH regulation and ion transport [13]. GO analysis of the predicted gene targets for differentially expressed miRNAs highlighted the functional categories that overlap with all the key processes during enamel maturation. For example, among the 18 genes significantly enriched in the category “endosome membrane” (Table 3), up-regulation of *Cftr* and *Lamp1* at both the mRNA and protein levels (maturation relative to secretory) have been shown previously [13, 40, 41]. Two other examples listed in Table 3, *Slc26a7* and Tfrc, have also been confirmed as being up-regulated at both the mRNA and protein levels (maturation relative to secretory) by our group (data not included). *Lamp1* and *Tfrc* are considered to be the potential ameloblast membrane-bound receptors for EMP debris [20, 40, 42–44], and are involved with trafficking between the plasma membrane and endosomal/lysosomal structures through associations with one or more adaptor protein complexes. CFTR functions as a regulator of pH during rapid crystal growth and is critical for completion of enamel mineralization [41, 45–47]. CFTR is expressed most highly in maturation-stage ameloblasts; furthermore, in *Cftr*-deficient animals that exhibit hypomineralized enamel, only the maturation-stage ameloblasts are structurally affected [41, 45–47]. Apart from these relatively well-studied genes, *Slc11a2* (listed in Table 3), which is involved in iron transport in epithelial tissues, is regulated by *Cd61* in maturation-stage ameloblasts [48]. Another gene of note, listed in the category of “cytokine activity” (Table 6), is *Wnt5a. Wnt5a*-induced cell death is crucial in determining the tooth size during murine tooth development [49]. In addition, enriched in the category “carboxylic acid transmembrane transporter activity” are 11 SLC gene family members (Table 1). The mRNA expression levels of Slc1a1, Slc6a8, Slc25a15, Slc26a1 [13] and the other solute carrier (SLC) gene family members (listed in Table 1) are significantly up-regulated in the enamel organ cells during enamel maturation, and highlight the importance of SLC-mediated chloride-bicarbonate exchange in enamel maturation. It is noteworthy that GO analysis of the miRNA-regulated genes indicated that 27 genes up-regulated during maturation stage genes were significantly enriched in the category of “calcium ion binding” (Table 5), while 11 genes down-regulated during maturation stage were also enriched in the category of “calcium ion transmembrane transporter activity” (Table 7). These data lend support to the complexity of genetic networks controlling enamel maturation, or even the whole process of amelogenesis.

Experimental validation would be required for the identification of miRNA target genes *in vivo* following computational prediction of miRNA and mRNA interaction. To date, there is still no consensus about the working schemes of experimental validation of miRNA targets *in vivo*. However, in addition to computational prediction, at least three criteria should be met before confirming the miRNA regulator for a given gene [50]: 1) miRNA/mRNA coexpression; 2) miRNA effect on target proteins; 3) miRNA effect on biological functions. While all of these criteria might not be met under all conditions, nevertheless it is advisable that as many be achieved as possible. In our efforts to validate the regulatory relations between *Lamp1* and miR-153, as well as between *Tfrc* and miR-31, we first demonstrated the coexpression of miRNAs and mRNAs of target genes during maturation-stage development. In the case of *Lamp1* and miR-153 there was an inverse relationship, with higher *Lamp1* mRNA levels and lower miR-153 levels noted in the maturation stage (Table 4). In the case of *Tfrc* and miR-31, the highest levels of expression of both were noted in maturation-stage amelogenesis (Additional files 1, 2 and 19). The interaction between the seeding sequence of the miRNA (miR-153 and miR-31) and the 3′-UTR of the target mRNA (*Lamp1* and *Tfrc*) was predicted by TargetScan, and the binding site of miR-31 to the mRNA of *Tfrc* was predicted to be highly conserved across vertebrates. The effects of miR-153 and miR-31 on target proteins (*Lamp1* and *Tfrc* respectively) was identified indirectly by the changes in luciferase reporter assays induced by exogenously introduced mimics and inhibitors of corresponding miRNAs into the host cells (Figures 7B and 8B). The experiments seeking to confirm the effects of these miRNAs on biological functions is not possible at this stage due to a lack of suitable animal models and/or appropriate organ culture systems. As part of our future approach to studying the role of miRNAs in amelogenesis, gain-of-function or loss-of-function studies of miRNAs should be considered in suitable primary organ cultures or with the development of appropriate animal models. A caveat about investigating the functional role of miRNAs is that there can be functional redundancy among miRNAs. For example, miR-21 and miR-31 facilitate invasion and metastasis of colon carcinoma cells by suppressing the same target TIAM1 in TGF-β signaling pathway [51]. In human natural regulatory T cell *FOXP3* expression is affected by both miR-21 and miR-31, although the regulation of miR-21 is indirect [52]. Thus loss-of-function of a single miRNA, especially the one of multi-gene family may not result in an aberrant phenotype [53].

Finally, it has been a widely accepted concept that gene expression regulation is controlled by various factors at protein and RNA levels [54]. Although the key role of miRNA regulation has been suggested in multiple biological processes and diseases, it cannot be the only contributor to the intrinsic complexity of regulatory networks. In our study, 1729 genes (transcripts) showed differential expression during maturation-stage tooth development. Among the 1729 genes, 299 up-regulated and 330 down-regulated genes were predicted to be regulated by the 59 differentially expressed miRNAs (Additional files 5 and 6). On the other hand, the remaining stably expressed miRNAs were predicted to be the regulators of 828 up-regulated and 513 down-regulated genes (out of 1729). These data suggested that genetic regulators other than miRNAs, such as transcription factors, must be dominant players in the processes of enamel maturation. In the IPA core analysis of the miRNA-regulated candidate gene lists (299 up-regulated and 330 down-regulated), the potential transcription factors were predicted (Additional files 15 and 16; Upstream Regulators). It is highly possible that miRNAs and transcription factors co-regulated the expression of these two groups of target genes, in forms of feed-forward loops (FFLs) and feedback loops (FBLs) [54–57].

## Conclusion

In conclusion, miRNAs are dynamically expressed when tooth development transitions from the secretory stage to the maturation stage, and the differentially expressed miRNAs likely play a role in the regulation of enamel maturation events by targeting genes involved in specific activities such as pH regulation, ion transport, endocytosis and apoptosis. The data presented here should help identify the roles of individual miRNAs in amelogenesis, and more generally help to clarify the potential roles of miRNA-centered regulatory mechanisms in mineralized tissues. Our data also suggest additional experiments to establish causal relationships between miRNA and mRNA levels in genetically modified cell culture and animal models are warranted. Recognition of miRNA-related regulation and the functions of corresponding target genes during enamel development may also shed light on clinical diagnosis and/or treatment of diseases such as amelogenesis imperfecta.

### Availability of supporting data

The data sets supporting the results of this article are included within the article and its additional files.

## Electronic supplementary material

Additional file 1: **Normalized miRNA expression scores, fold changes and P values.** (XLSX 297 KB)

Additional file 2: **Normalized (log) mRNA transcripts expression scores, fold changes and** ***P*** **values.** (XLSX 5 MB)

Additional file 3: **Number of differentially expressed mRNAs with a 5% FDR and different fold changes.** (XLSX 9 KB)

Additional file 4: **Volcano plot depicting the miRNA expression data.** the relationships between fold changes of miRNA expression (X-axis) and adjusted p values (Y-axix) are provided. Differentially expressed miRNAs (maturation/secretory, Fold Changes ≥ 1.2/≤-1.2, FDR <0.05 are highlighted in red, while the non-differentially expressed miRNAs are colored in blue. (PPTX 2 MB)

Additional file 5: **Up-regualted miRNAs at maturation-stage tooth development relative secretory-stage and their predicted gene targets expressed in opposite direction.** (XLSX 20 KB)

Additional file 6: **Down-regualted miRNAs at maturation-stage tooth development relative secretory-stage and their predicted gene targets expressed in opposite direction.** (XLSX 21 KB)

Additional file 7: **Non-differentially expressed miRNAs between secretory- and maturation-stage tooth development and their predicted target that are up-regulated during maturation-stage (relative to secretory-stage).** (XLS 509 KB)

Additional file 8: **Non-differentially expressed miRNAs between secretory- and maturation-stage tooth development and their predicted target that are down-regulated during maturation-stage (relative to secretory-stage).** (XLS 354 KB)

Additional file 9: **Flow chart depicting the strategies used to select targets for differentially (Observed) and non-differentially (Baseline) expressed miRNAs.** Approximately 5.8% (629/10,786) of the candidate target genes were differentially expressed in the expected direction in our analysis. By comparison, approximately 8.1% of candidate target mRNAs (1341 total: 828 up-regulated and 513 down-regulated) were identified to be the potential targets for the stably expressed miRNAs. (PPTX 53 KB)

Additional file 10: **Up-regualted miRNAs at maturation-stage tooth development relative secretory-stage (FC > 5) and their predicted gene targets expressed in the same direction.** (XLSX 11 KB)

Additional file 11: **Gene Ontology analysis of the 330 up-regulated transcripts (maturation relative to secretory) that are predicted to be regulated by the 20 down-regulated miRNAs (maturation relative to secretory).** (XLSX 987 KB)

Additional file 12: **Gene Ontology analysis of the 299 down-regulated transcripts (maturation relative to secretory) that are predicted to be regulated by the 39 up-regulated miRNAs (maturation relative to secretory).** (XLSX 667 KB)

Additional file 13: **KEGG analysis of the 330 up-regulated transcripts (maturation relative to secretory) that are predicted to be regulated by the 20 down-regulated miRNAs (maturation relative to secretory).** (XLSX 68 KB)

Additional file 14: **KEGG analysis of the 299 down-regulated transcripts (maturation relative to secretory) that are predicted to be regulated by the 39 up-regulated miRNAs (maturation relative to secretory).** (XLSX 31 KB)

Additional file 15: **IPA core analysis of differentially expressed miRNAs and their predicted gene target candidates (with 140 molecules involved per network interaction).** (XLSX 278 KB)

Additional file 16: **IPA core analysis of differentially expressed miRNAs and their predicted gene target candidates (with 70 molecules involved per network interaction).** (XLSX 250 KB)

Additional file 17: **IPA core analysis of down-regulated miRNAs and their validated predicted gene target candidates (with 140 molecules involved per network interaction).** (XLSX 184 KB)

Additional file 18: **IPA core analysis of down-regulated miRNAs and their validated predicted gene target candidates (with 140 molecules involved per network interaction).** (XLSX 177 KB)

Additional file 19: **IPA Gene Network Analysis.** Top gene networks for differentially expressed miRNAs (maturation/secretory) and their validated predicted gene target candidates were generated by IPA to show their directed interactions, with 140 **(Panels A-C)** and 70 **(Panels D-F)** molecules involved in each network. (PPTX 11 MB)

Additional file 20: **IPA Gene Network Analysis.** Top gene networks for down-regulated miRNAs (maturation/secretory) and their validated predicted gene target candidates were generated by IPA to show their directed interactions, with 140 **(Panels A-C)** and 70 **(Panels D-F)** molecules involved in each network. (PPTX 10 MB)

Additional file 21: **IPA Gene Network Analysis.** Top gene networks for up-regulated miRNAs (maturation/secretory) and their validated predicted gene target candidates were generated by IPA to show their directed interactions, with 140 **(Panels A-C)** and 70 **(Panels D-F)** molecules involved in each network. (PPTX 10 MB)

## Abbreviations

AI: Amelogenesis imperfecta
B-H: Benjamini Hochberg
DEGs: Differentially expressed genes
ECM: Extracellular matrix
FBS: Fetal bovine serum
FC: Fold changes
FDR: False discovery rate
PGS: Partek® genomics suite
GO: Gene ontology
IACUC: Institutional Animal Care and Use Committee
IPA: Ingenuity pathway analysis
KEGG: Kyoto Encyclopedia of genes and genomics
LNA-DIG: Locked nucleic acid-digoxygenin
NCBI: National Center for Biotechnology Information
PFA: Paraformaldehyde
qPCR: quantitative Polymerase chain reaction
RNU6-2: U6 Small nuclear 2
SCGC: Southern California Genotyping Consortium
3′-UTR: Three prime untranslated region.
