# Supplementary material for: Genome-wide analysis of miRNA and mRNA transcriptomes during amelogenesis
Source: BMC Genomics. 2014 Nov 19;15(1):998. doi: 10.1186/1471-2164-15-998 (PMC4254193; doi:10.1186/1471-2164-15-998)
Supplement: Supplementary file 4 — Additional file 4: Volcano plot depicting the miRNA expression data. the relationships between fold changes of miRNA expression (X-axis) and adjusted p values (Y-axix) are provided. Differentially expressed miRNAs (maturation/secretory, Fold Changes ≥ 1.2/≤-1.2, FDR <0.05 are highlighted in red, while the non-differentially expressed miRNAs are colored in blue. (PPTX 2 MB) [file 12864_2014_6698_MOESM4_ESM.pptx]

## Slide 1
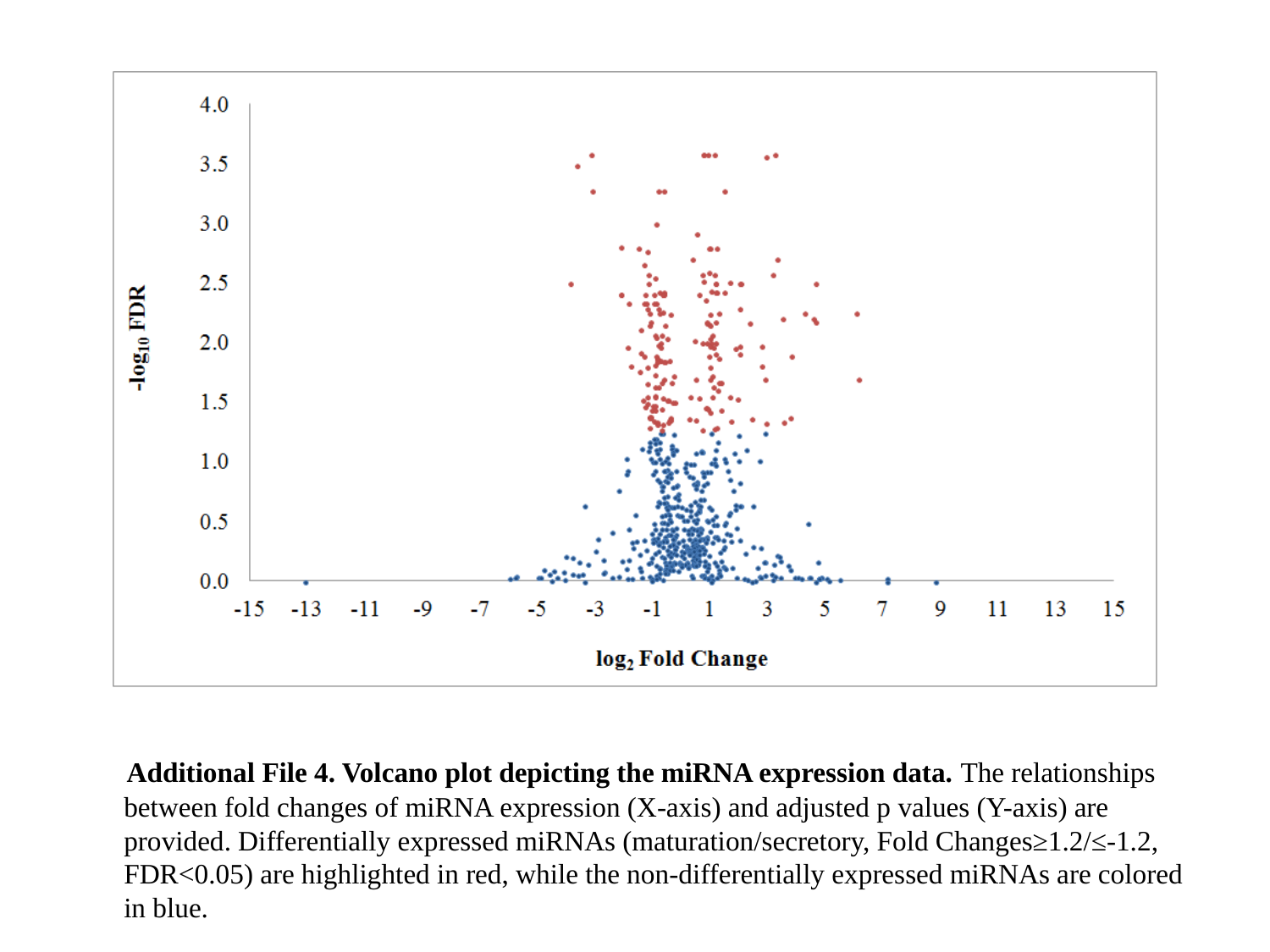

Additional File 4. Volcano plot depicting the miRNA expression data. The relationships between fold changes of miRNA expression (X-axis) and adjusted p values (Y-axis) are provided. Differentially expressed miRNAs (maturation/secretory, Fold Changes≥1.2/≤-1.2, FDR<0.05) are highlighted in red, while the non-differentially expressed miRNAs are colored in blue.
