# Supplementary material for: Genome-wide analysis of miRNA and mRNA transcriptomes during amelogenesis
Source: BMC Genomics. 2014 Nov 19;15(1):998. doi: 10.1186/1471-2164-15-998 (PMC4254193; doi:10.1186/1471-2164-15-998)
Supplement: Supplementary file 9 — Additional file 9: Flow chart depicting the strategies used to select targets for differentially (Observed) and non-differentially (Baseline) expressed miRNAs. Approximately 5.8% (629/10,786) of the candidate target genes were differentially expressed in the expected direction in our analysis. By comparison, approximately 8.1% of candidate target mRNAs (1341 total: 828 up-regulated and 513 down-regulated) were identified to be the potential targets for the stably expressed miRNAs. (PPTX 53 KB) [file 12864_2014_6698_MOESM9_ESM.pptx]

## Slide 1
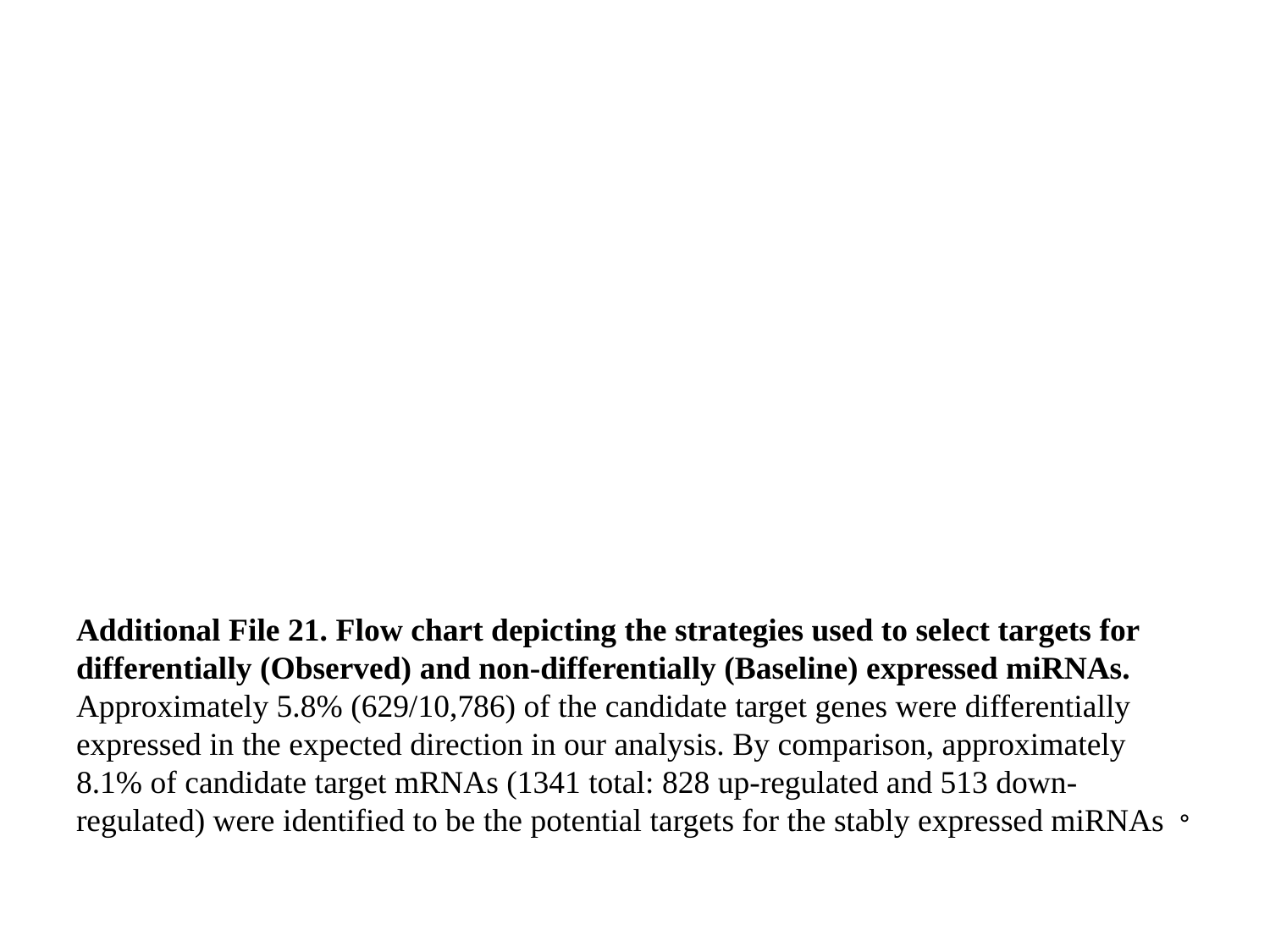

Additional File 21. Flow chart depicting the strategies used to select targets for differentially (Observed) and non-differentially (Baseline) expressed miRNAs. Approximately 5.8% (629/10,786) of the candidate target genes were differentially expressed in the expected direction in our analysis. By comparison, approximately 8.1% of candidate target mRNAs (1341 total: 828 up-regulated and 513 down-regulated) were identified to be the potential targets for the stably expressed miRNAs。

## Slide 2
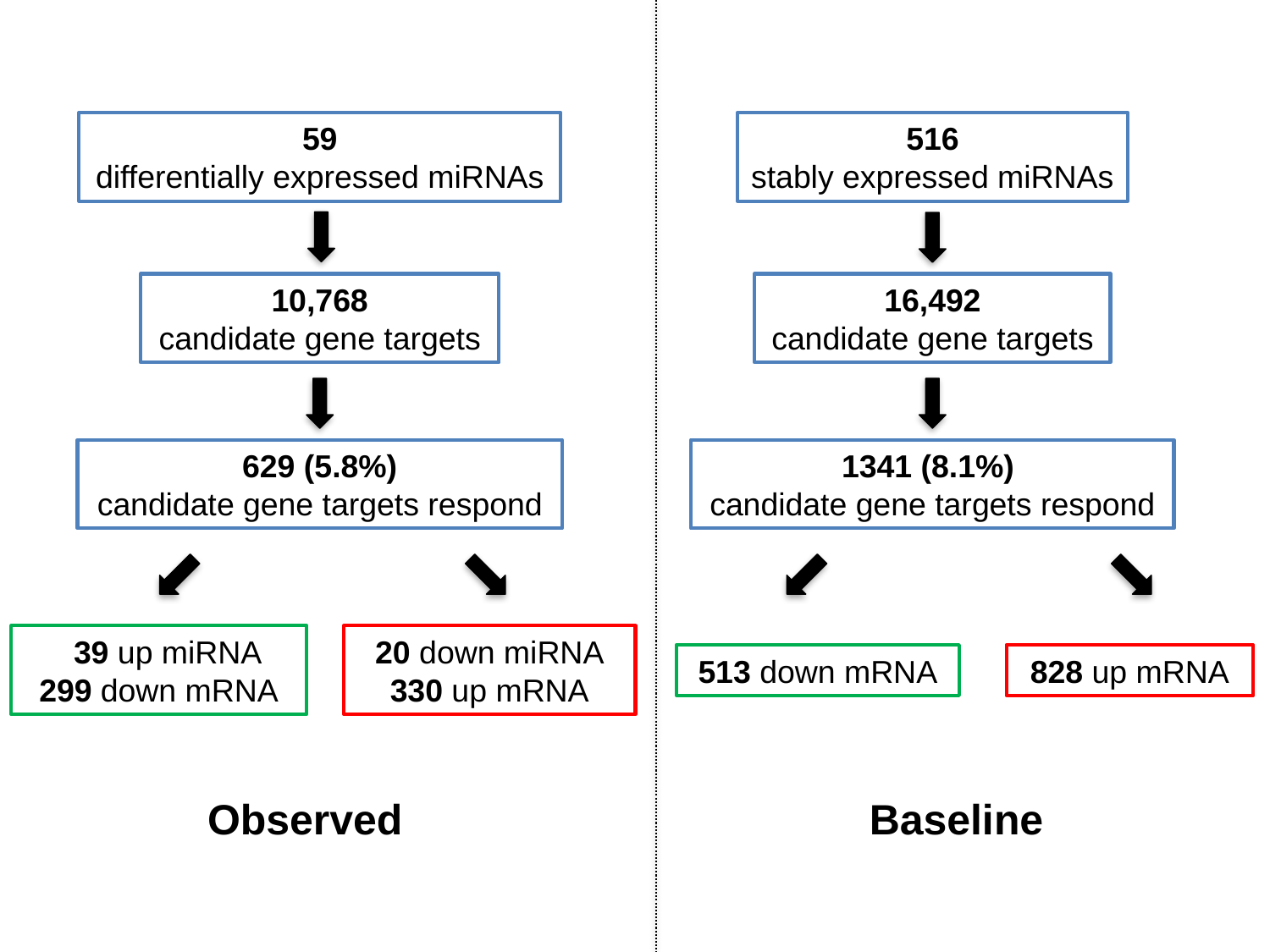

59
differentially expressed miRNAs
516
stably expressed miRNAs
10,768
candidate gene targets
16,492
candidate gene targets
629 (5.8%)
candidate gene targets respond
1341 (8.1%)
candidate gene targets respond
 39 up miRNA
299 down mRNA
20 down miRNA
330 up mRNA
513 down mRNA
828 up mRNA
Observed
Baseline
