# Supplementary material for: Genome-wide analysis of miRNA and mRNA transcriptomes during amelogenesis
Source: BMC Genomics. 2014 Nov 19;15(1):998. doi: 10.1186/1471-2164-15-998 (PMC4254193; doi:10.1186/1471-2164-15-998)
Supplement: Supplementary file 20 — Additional file 20: IPA Gene Network Analysis. Top gene networks for down-regulated miRNAs (maturation/secretory) and their validated predicted gene target candidates were generated by IPA to show their directed interactions, with 140 (Panels A-C) and 70 (Panels D-F) molecules involved in each network. (PPTX 10 MB) [file 12864_2014_6698_MOESM20_ESM.pptx]

## Slide 1
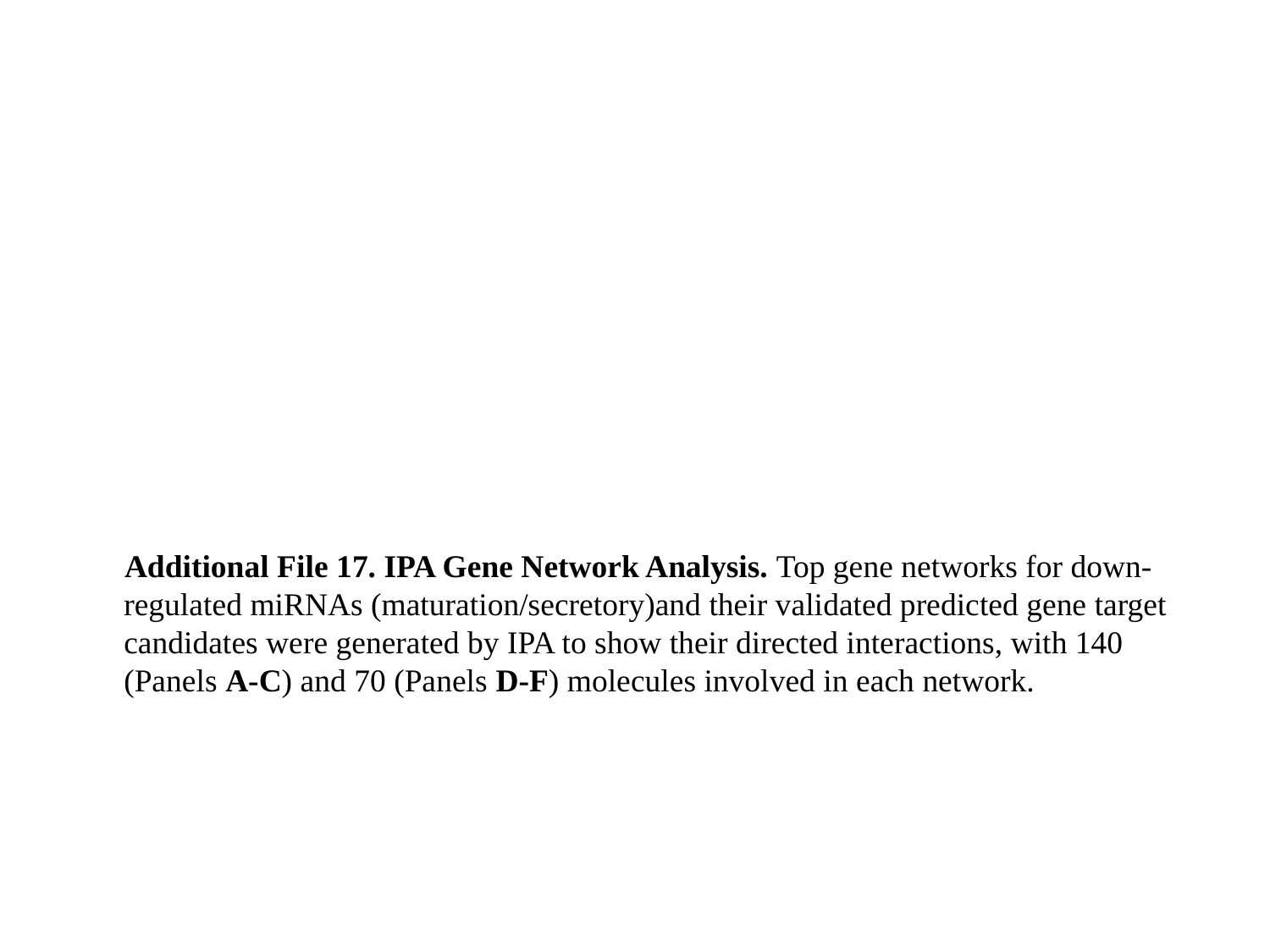

Additional File 17. IPA Gene Network Analysis. Top gene networks for down-regulated miRNAs (maturation/secretory)and their validated predicted gene target candidates were generated by IPA to show their directed interactions, with 140 (Panels A-C) and 70 (Panels D-F) molecules involved in each network.

## Slide 2
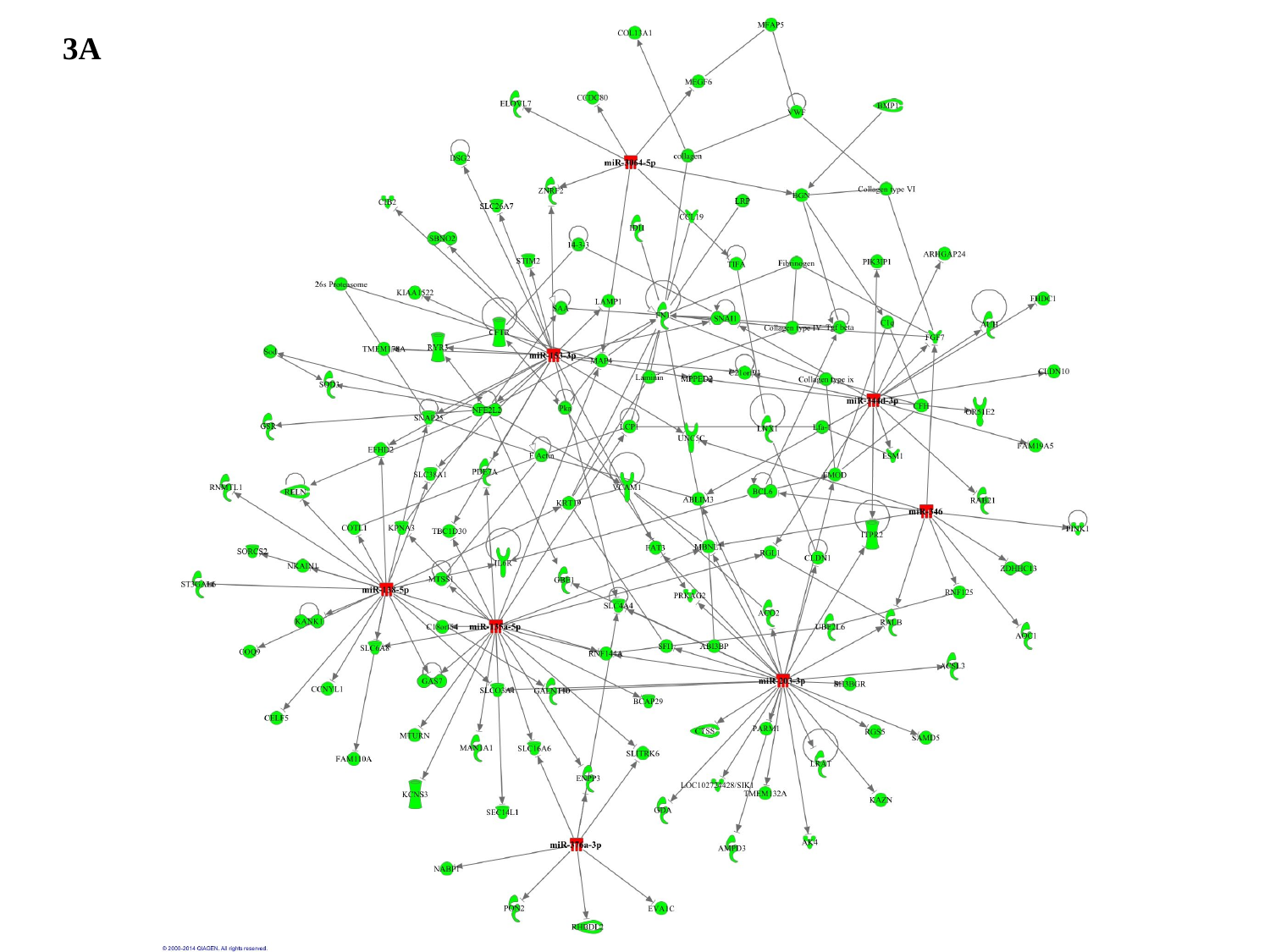

3A

## Slide 3
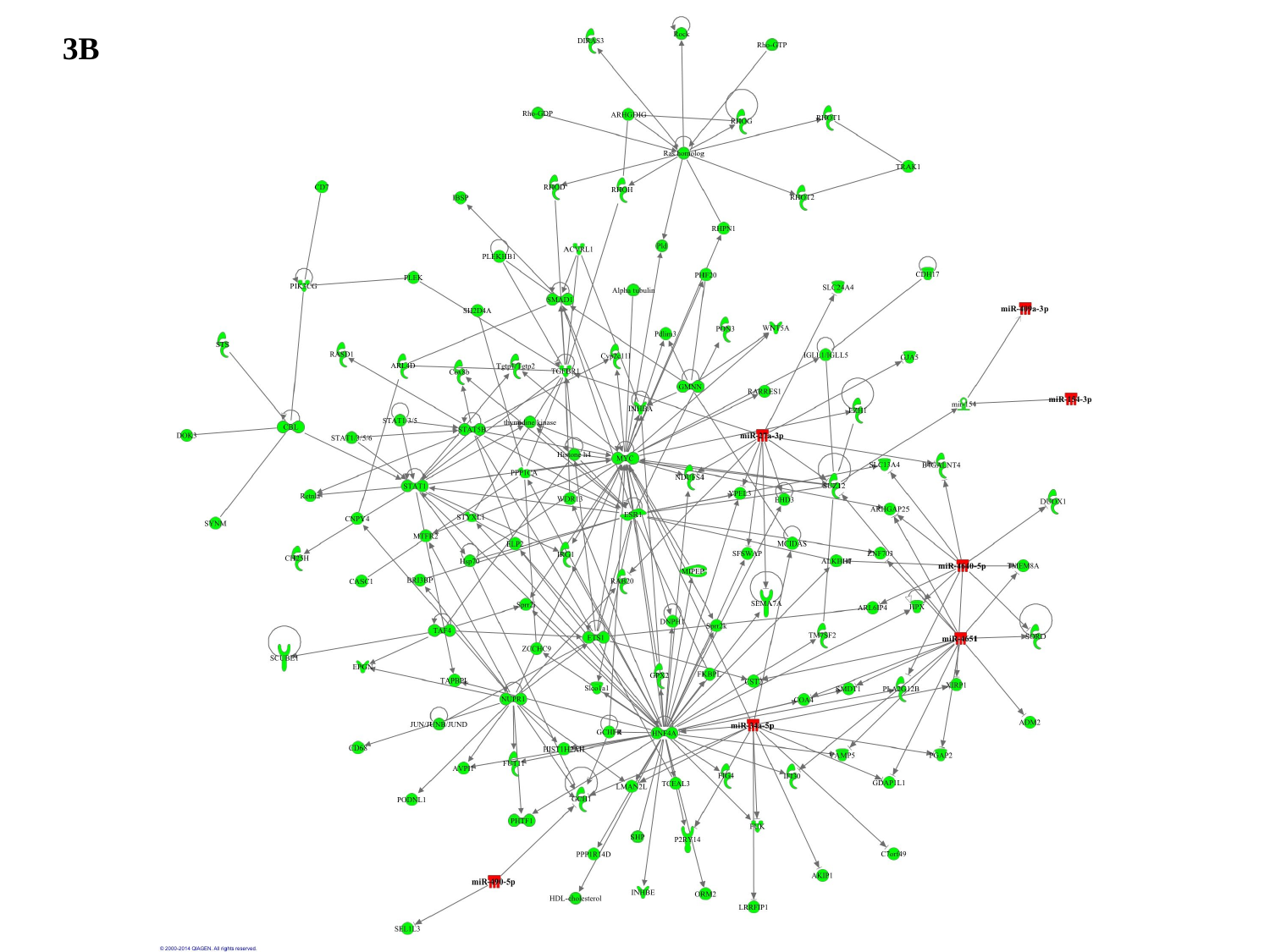

3B

## Slide 4
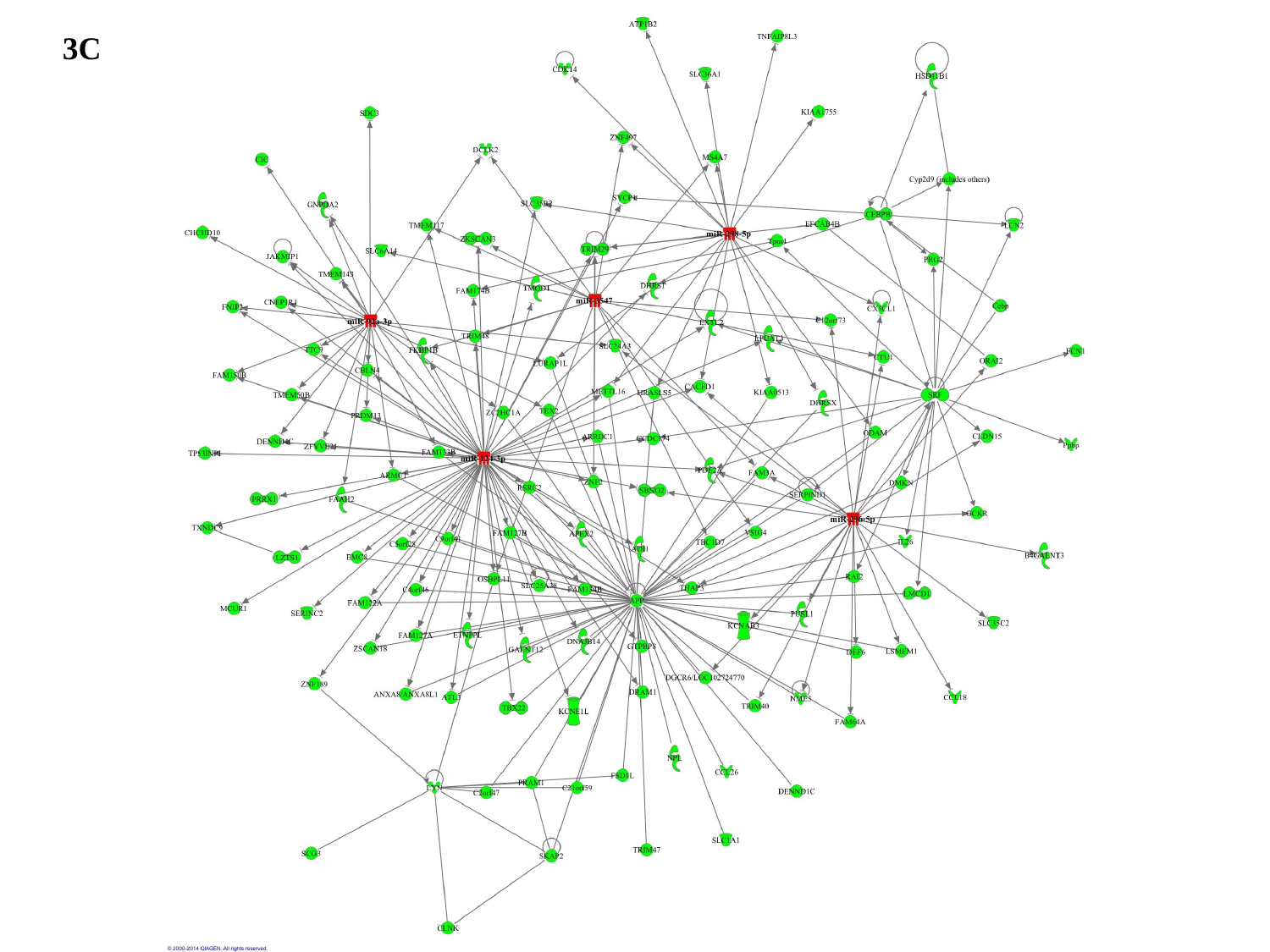

3C

## Slide 5
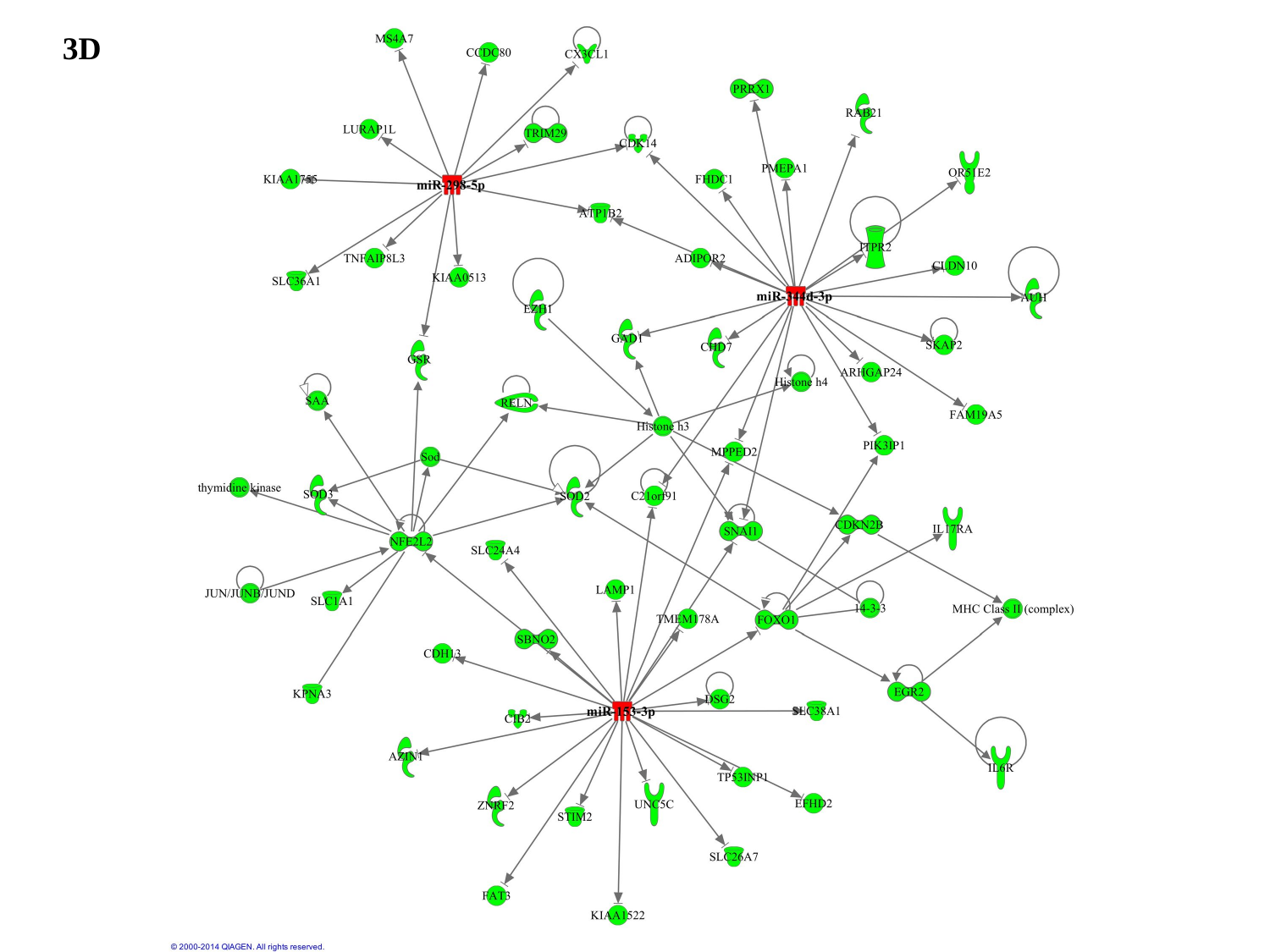

3D

## Slide 6
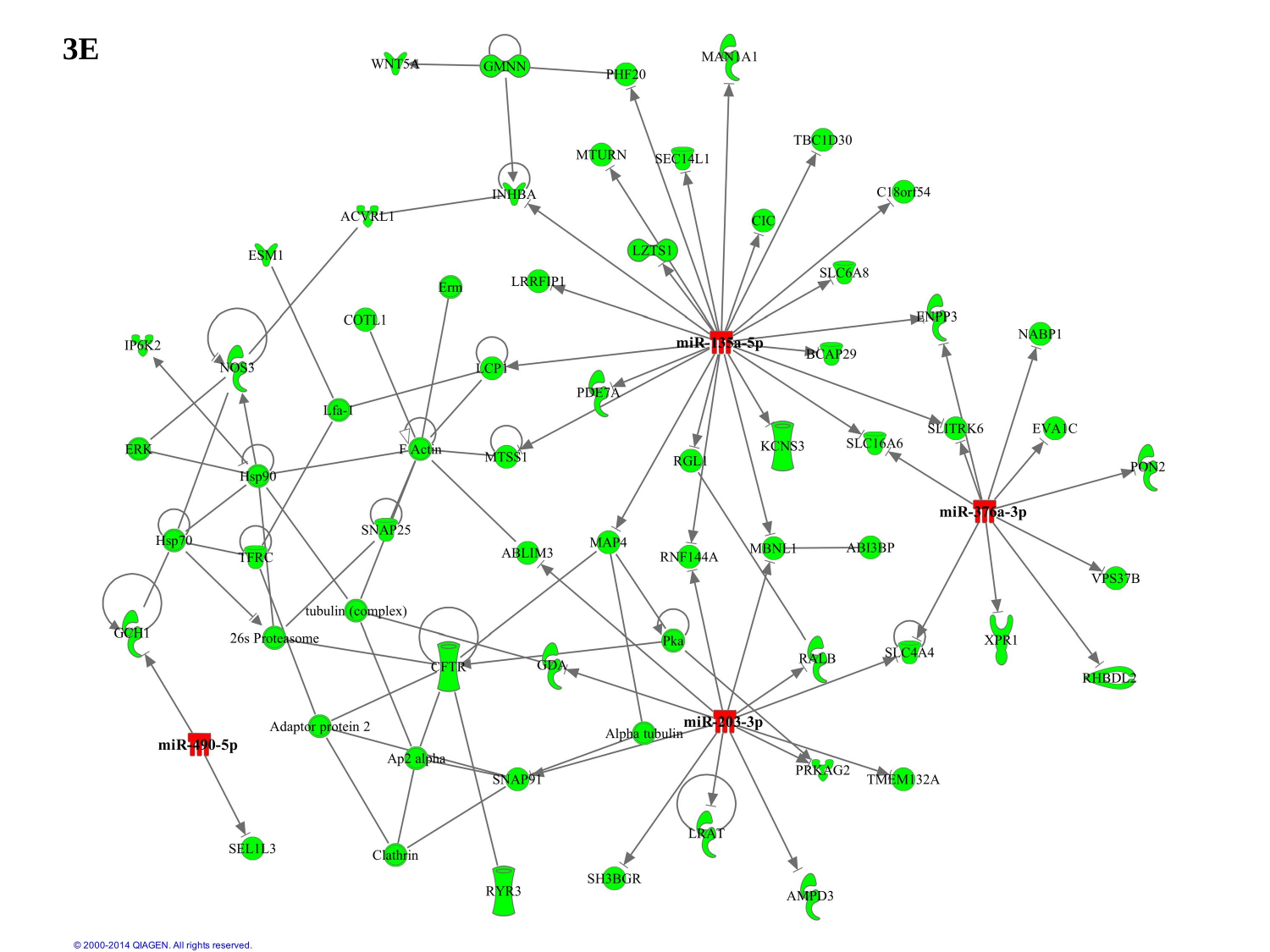

3E

## Slide 7
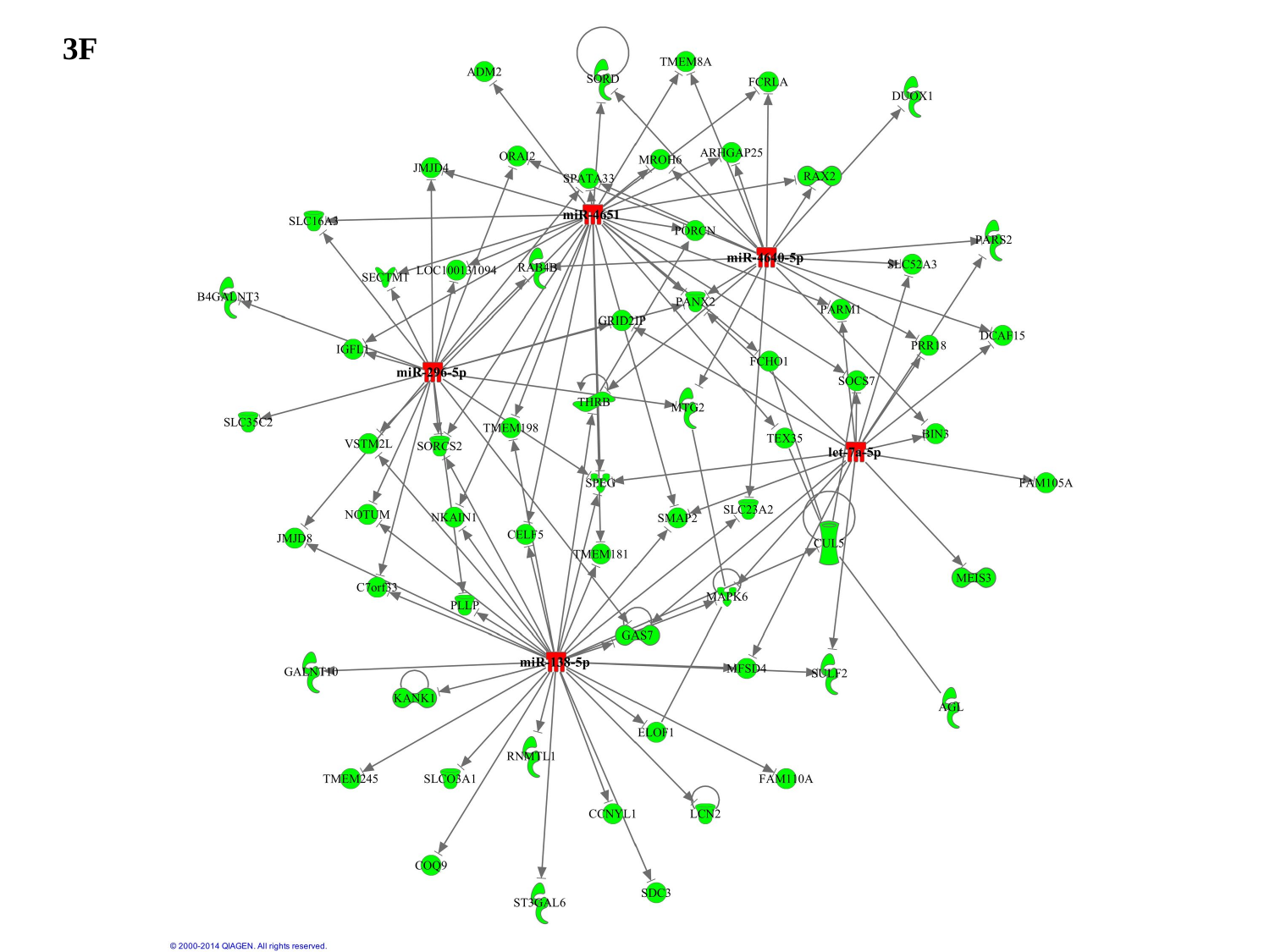

3F
